# Systems metabolic engineering of a plasmid-free *Escherichia coli* cell factory for high-level cytidine production

**DOI:** 10.1016/j.synbio.2026.09.003

**Published:** 2026-09-22

**Authors:** Runze Zhao, Changgeng Li, Chaoye Xu, Zhichao Chen, Haibo Xiong, Zichen Yu, Tang'en Shi, Lanxiao Li, Meng Yuan, Ling Ma, Siyu Zhao, Hongyu Deng, Qingyang Xu

**Affiliations:** a College of Biotechnology, Tianjin University of Science & Technology, Tianjin, 300457, PR China; b Key Laboratory of Industrial Fermentation Microbiology of the Ministry of Education, Tianjin University of Science & Technology, Tianjin, 300457, PR China; c State Key Laboratory of Green Papermaking and Resource Recycling, Qilu University of Technology (Shandong Academy of Science), Jinan, 250353, PR China

**Keywords:** Cytidine, Metabolic engineering, *Escherichia coli*, Channel engineering, Dynamic regulation, Respiratory chain engineering

## Abstract

Cytidine is an important precursor for nucleoside pharmaceuticals, but its fermentative production is constrained by competing degradation, poor selectivity of pyrimidine flux, insufficient precursor supply, and energy-redox imbalance. In this study, a plasmid-free *E. coli* cell factory was constructed through systems metabolic engineering. Sequential deletion of cytidine degradation, salvage, and precursor-consuming pathways enabled initial cytidine accumulation. The conversion from UMP to CMP was strengthened by introducing feedback-deregulated *pyrH*^D93A^ and *pyrG*^D160E, E162A, E168K^_cgl_ mutants and *nudG*, while the heterologous expression of *PHM8*_sce_ improved CMP-to-cytidine conversion selectivity. Scaffold-based assembly of PyrF, PyrH^D93A^, NudG, and PHM8_sce_ using CipA/CipB further improved local pathway channeling and reduced uridine formation by 98.70%. To reinforce upstream flux, de novo UMP biosynthesis was reconstructed with the *B. subtilis* pyr operon, PRPP synthesis was enhanced, and nitrogen-donor and pyrimidine-ring precursor supply was improved through *glnA*, *ppc*, and *aspC* expression. Finally, an ATP-responsive *ydaO* motif regulatory module was used to dynamically control respiratory-chain and oxygen-utilization modules, coordinating ATP supply with NAD^+^ regeneration. The final strain cyt 23 produced 96.20 g/L cytidine in 50 h in a 5 L fed-batch bioreactor, with a glucose conversion yield of 0.38 g/g and productivity of 1.92 g/(L·h), representing the highest reported titer, yield and productivity for microbial cytidine production to date. This work provides an integrated strategy for high-level microbial cytidine production and nucleoside cell-factory development.

## Highlights

• Systems metabolic engineering coordinated cytidine flux and precursor supply.
• CipA/CipB-based channeling engineering reduced uridine formation by 98.57%.
• ATP-responsive *ydaO* motif balanced energy generation and NAD ^+^ regeneration.
• Cyt 23 produced 96.20 g/L cytidine, the highest reported microbial titer to date.

## Graphical abstract

**Figure:**
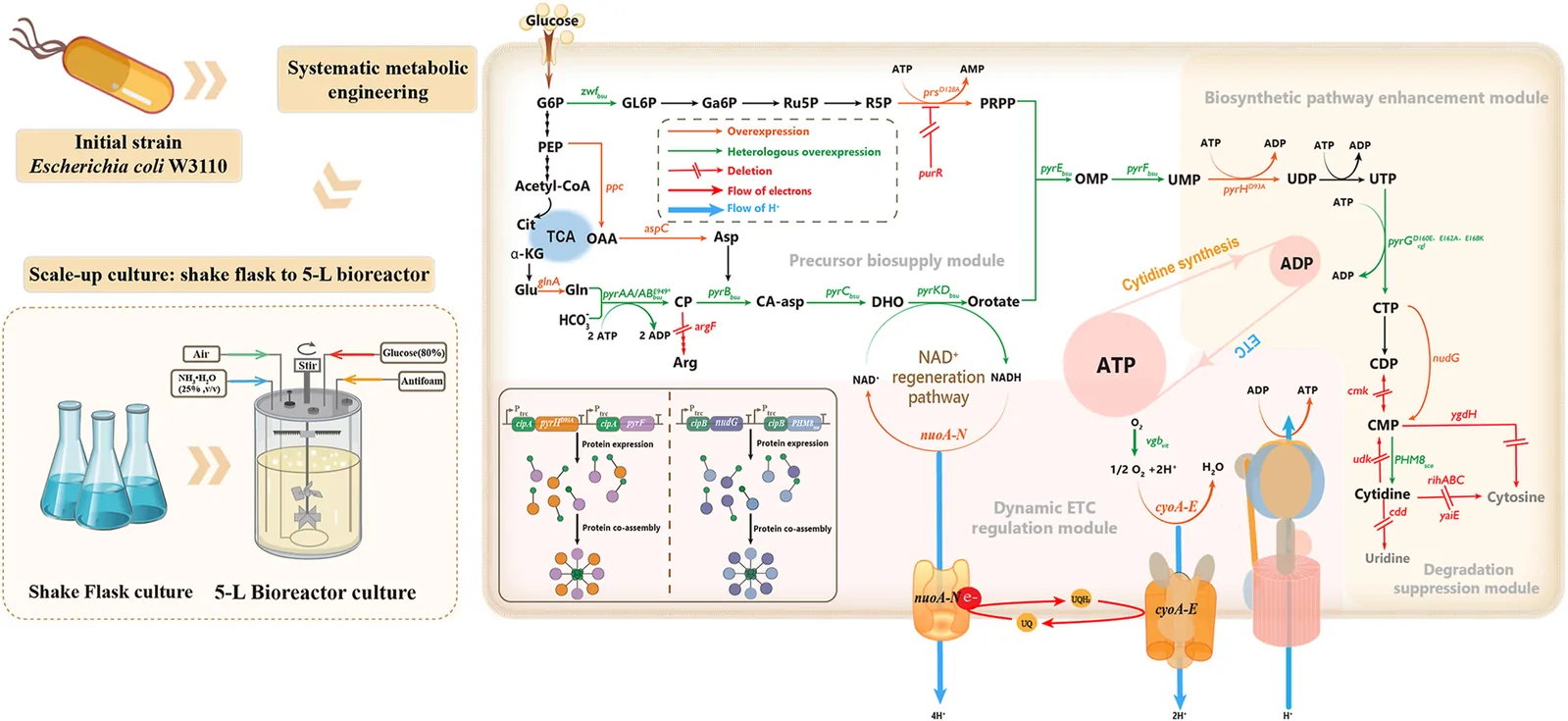

## 1 Introduction

Cytidine is a pyrimidine nucleoside that results from joining cytosine to ribose via a β-N1-glycosidic bond. It serves as a fundamental structural component of ribonucleic acid (RNA) and an essential building block for life; at the same time, it is a necessary chiral starting material in the synthesis of many high-value nucleoside analogues [1,2]. Cytidine and its derivatives are used to prepare many antiviral and anticancer drugs, such as cytarabine, gemcitabine, lamivudine and sofosbuvir [3,4]. The expanding development of nucleoside analogue therapeutics has increased interest in reliable and sustainable cytidine supply routes [5].

Traditionally, cytidine has been produced by chemical synthesis or by enzymatic and chemical hydrolysis of yeast RNA; both methods generally have a low yield, a complex purification process, and use hazardous reagents [6]. In contrast, de novo fermentative synthesis from renewable feedstocks offers a potentially more sustainable route under mild aqueous conditions and is compatible with scalable cell-factory engineering [7]. Because of its simple genetic system, relatively clear metabolic network and abundant editing tools, *Escherichia coli* (*E. coli*) has been selected as a production host for nucleosides [8].

Several strategies have been proposed to enhance cytidine production by *E. coli*. Early work used random mutagenesis and resistance-based screening, or rationally modified single nodes, such as deletion of uridine-cytidine kinase (encoded by *udk*) to block cytidine re-uptake and attenuation of cytidine deaminase (encoded by *cdd*) to prevent degradation [9,10]; these approaches were also combined with atmospheric and room-temperature plasma (ARTP) mutagenesis to increase synthetic capacity [11]. To overcome feedback inhibition, feedback-resistant mutants of aspartate transcarbamoylase (encoded by *pyrB*/*pyrI*) and carbamoyl phosphate synthetase (encoded by *carAB*) have been introduced to eliminate negative regulation of upstream metabolic flux by uridine triphosphate (UTP) and cytidine triphosphate (CTP) [12]. In terms of precursor reinforcement, co-expression of *prs*, *zwf* and *gnd* increases the supply of phosphoribosyl pyrophosphate (PRPP) and reduced NADPH [13], enhanced provision of aspartate and glutamine replenishes the nitrogen source for the pyrimidine ring [14], and overexpression of a permease and a nucleoside transporter from *Bacillus amyloliquefaciens* promotes cytidine export [15]. More recently, systematic and modular engineering strategies have further improved cytidine biosynthesis by reconstructing the pyrimidine pathway, regulating carbon metabolism and cofactor supply, or combining transcriptomics and metabolomics to identify limiting genes [[16], [17], [18], [19], [20], [21]]. Collectively, these studies have improved cytidine production by reducing degradation, relieving feedback inhibition, reinforcing precursor supply, and promoting product export. However, most strategies have focused on individual nodes rather than the coordinated redistribution of pathway flux, precursor availability, and cellular energy metabolism.

Although these efforts have gradually increased the scale of cytidine production from milligrams to grams, three deeper bottlenecks have not been addressed systematically. The first is severe carbon loss: The enzymatic system of the cytidine pathway is complex, the route is long, and most of the enzymes involved have limited specificity; thus, byproducts such as uridine and uracil are readily generated during strain engineering, leading to significant loss of carbon and precursors. The second is limited energy supply: de novo cytidine synthesis is very energy-intensive, as the formation of uridine monophosphate (UMP) and PRPP, and the assembly of the pyrimidine ring all require a large amount of adenosine triphosphate (ATP), but almost no existing research actively regulates energy metabolism or respiratory chain efficiency, so insufficient ATP supply has become an unseen bottleneck limiting high-intensity synthesis. The third is cofactor imbalance; reconstruction of the cytidine pathway often disrupts intracellular redox homeostasis and thus inhibits both cell proliferation and product accumulation. More significantly, most of the existing research focuses on the local optimization of isolated bottlenecks and fails to address, in an integrated and coordinated manner, the intertwined and mutually constraining relationships among precursor supply, ATP supply, cofactor balance and byproduct formation. These limitations indicate that efficient cytidine biosynthesis requires more than pathway amplification. A productive cell factory must simultaneously reduce carbon loss to competing nucleosides, maintain sufficient UMP and PRPP precursor supply, and support the ATP and redox demands imposed by de novo pyrimidine biosynthesis.

To address these constraints, this study constructed a plasmid-free *E. coli* cytidine producer through coordinated systems metabolic engineering (Fig. 1). Unlike previous studies that mainly focused on individual bottlenecks, such as cytidine degradation, feedback regulation, precursor supply, or transport, this work systematically integrated multi-layer engineering strategies to enhance cytidine biosynthesis. Cytidine degradation, salvage, and competing cytidine monophosphate (CMP) consumption were first attenuated to enable product accumulation. The UMP-to-cytidine route was then reinforced by relieving feedback inhibition, improving CMP-to-cytidine selectivity, and organizing key enzymes through scaffold-mediated channeling. Upstream precursor supply was further expanded by reconstructing de novo UMP biosynthesis and strengthening PRPP, nitrogen-donor, and pyrimidine-ring precursor availability. Finally, an ATP-responsive regulatory module was used to dynamically coordinate respiratory-chain activity, oxygen utilization, ATP supply, and NAD^+^ regeneration. By integrating pathway optimization, enzyme organization, precursor reinforcement, and energy-redox regulation into a single chromosomal platform, this study established a stable and high-performance cytidine-producing strain and offers a transferable strategy for microbial nucleoside production.

**Fig. 1:**
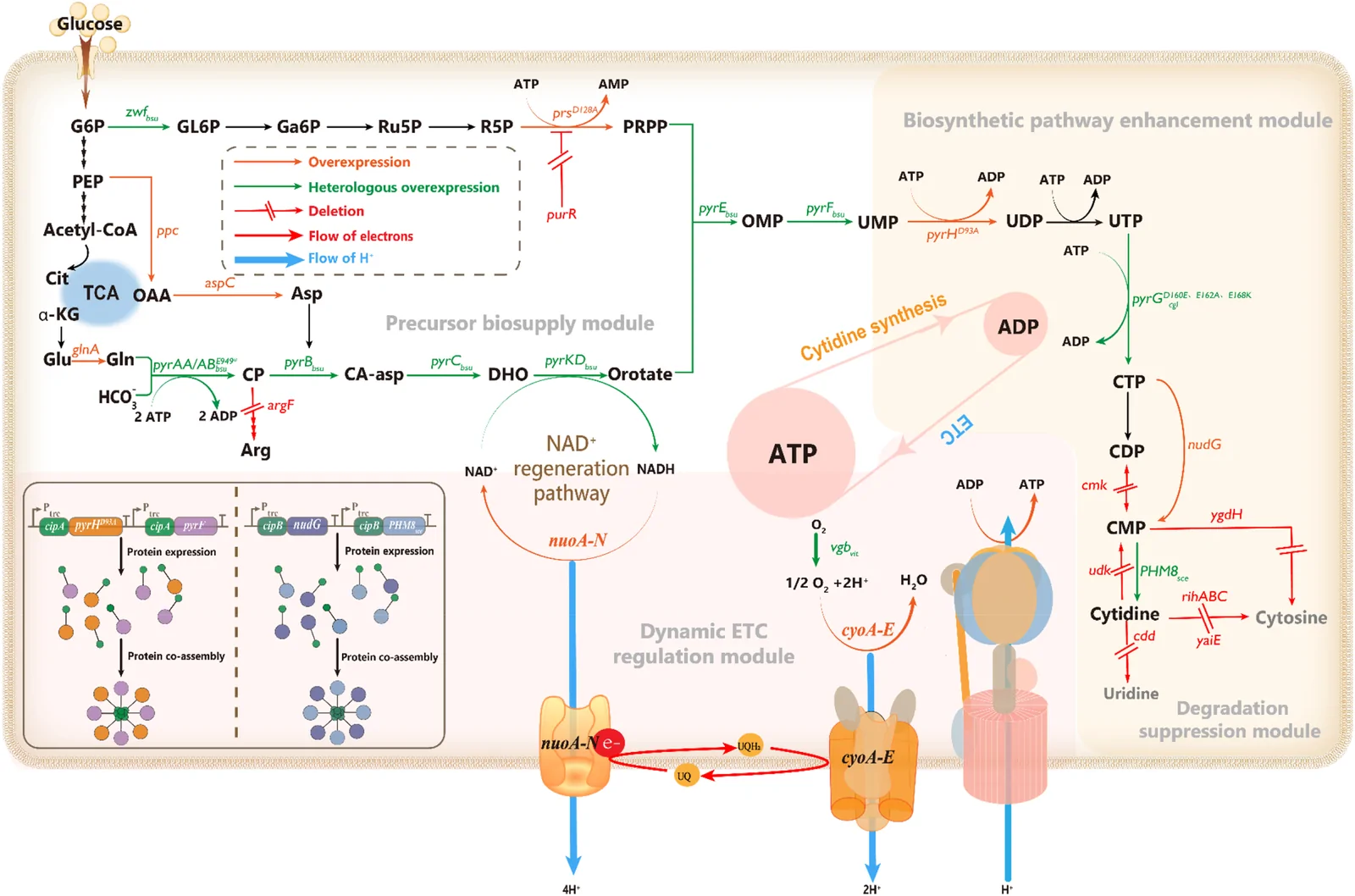
Metabolic pathways involved in cytidine biosynthesis in *E. coli.* and the metabolic engineering strategies employed in this study to improve cytidine production. Orange arrows indicate overexpressed native genes, green arrows indicate overexpressed heterologous genes, red arrows with double slashes indicate deleted genes, red arrows indicate electron flow, and blue arrows indicate H^+^ flow. G6P, glucose-6-phosphate; GL6P, glucono-1,5-lactone-6-phosphate; Ga6P, 6-phosphogluconate; Ru5P, ribulose-5-phosphate; R5P, ribose-5-phosphate; PRPP, 5′-phosphoribosyl pyrophosphate; PEP, phosphoenolpyruvate; acetyl-CoA, acetyl coenzyme A; Cit, citrulline; α-KG, α-ketoglutarate; OAA, oxaloacetate; Asp, aspartate; TCA, tricarboxylic acid cycle; Glu, glutamate; Gln, glutamine; CP, carbamoyl phosphate; CA-Asp, carbamoyl aspartate; DHO, dihydroorotate; OMP, orotidine 5′-monophosphate; UMP, uridine monophosphate; UDP, uridine diphosphate; UTP, uridine triphosphate; CTP, cytidine triphosphate; CDP, cytidine diphosphate; CMP, cytidine monophosphate; ETC, electron transport chain, also known as the respiratory chain.

## 2 Materials and methods

### 2.1 Strains and plasmids

All strains and plasmids used in this study are listed in Table S1 of the Supplementary Materials. *E. coli* W3110 served as the parental strain for all genetic modifications, and *E. coli* DH5α was used for plasmid construction and cloning. Plasmid pTrc99a was used to test the regulatory effect of the *ydaO* motif. Plasmids pREDCas9 and pGRB formed the clustered regularly interspaced short palindromic repeats/CRISPR-associated protein 9 (CRISPR/Cas9)-mediated genome editing system [22].

### 2.2 Acquisition of target genes and construction of recombinant plasmids

All primers in this study (Table S2) were provided by GENEWIZ Biotechnology Co., Ltd. Endogenous *E. coli* target genes were amplified using *E. coli* W3110 DNA as the template. Using *Bacillus subtilis* (*B. subtilis*) F126 genomic DNA as the template, we amplified the *B. subtilis*-derived *pyrB*-*pyrC*-*pyrAA*-*pyrAB*^E949^*-*pyrK*-*pyrD*-*pyrF*-*pyrE* cluster, *zwf* and the *ydaO* motif. *PHM8*_*sce*_ from *Saccharomyces cerevisiae* (*S. cerevisiae*), *vgb*_*vit*_ from *Vitreoscilla*, and *cipA* and *cipB* from *Photorhabdus luminescens* (Table S3) were codon-optimized and chemically synthesized by GENEWIZ Biotechnology Co., Ltd. Using amplification-based mutagenesis and *Corynebacterium glutamicum* (*C. glutamicum*) genomic DNA as the template, the mutant gene *pyrG* ^D160E, E162A, E168K^_cgl_ from *C. glutamicum* was obtained.

All mutant genes examined here were generated by amplification-based mutagenesis of the corresponding genomic DNA, as illustrated by the construction of *E. coli pyrH* ^D93A^. The upstream and downstream mutant fragments *pyrH*-up and *pyrH*-dw were first amplified from *E. coli* W3110 genomic DNA using the primer pairs *pyrH*-trc-UP-S/*pyrH*-UP-A and *pyrH*-DW-S/*pyrH*-DW-trc-A, respectively; primers *pyrH*-UP-A and *pyrH*-DW-S contained the mutated residue D93A and a shared homologous sequence. The two fragments were then purified and mixed for overlap-extension polymerase chain reaction (PCR) with primers *pyrH*-trc-UP-S and *pyrH*-DW-trc-A to obtain *pyrH* ^D93A^.

Plasmid pTrc99a-*ydaO*-*gfp* was constructed as follows: target fragments *ydaO*-1 and *gfp*-1 were amplified by PCR from the *ydaO* motif and *gfp* using primers *ydaO*-trc-S/*ydaO*-*gfp*-A and *ydaO*-*gfp*-S/*gfp*-trc-A, respectively, and the 3′ end of the former was homologous to the 5′ end of the latter. The linearized pTrc99a backbone was prepared using primers pTrc99a-XZ-1 and pTrc99a-XZ-2, and both ends contained homologous sequences for the *ydaO* and *gfp* fragments. Finally, recombinant plasmid pTrc99a-*ydaO*-*gfp* was constructed by homologous recombination between the target fragments and the linearized vector.

### 2.3 Strain construction and genome editing

Target genes were inserted into or deleted from *E. coli* W3110 using CRISPR/Cas9 [23]. Deletion of *cdd* is described as a representative example. Plasmid pGRB-*cdd* was first constructed: a double-stranded DNA (dsDNA) fragment containing sequences homologous to the pGRB backbone was synthesized by annealing primers pGRB-*cdd*-up and pGRB-*cdd*-dw, and the pGRB vector was linearized by PCR primers pGRB-S and pGRB-A; ligation of the dsDNA fragment into the linearized vector yielded recombinant plasmid pGRB-*cdd*. Donor DNA (DNA-*cdd*) was then prepared by amplifying the upstream and downstream homologous arms flanking *cdd* with primer pairs *cdd*-QC-1/cdd-QC-2 and *cdd*-QC-3/cdd-QC-4, respectively, followed by fusion of the two fragments through overlap-extension PCR. The resulting DNA-*cdd* and plasmid pGRB-*cdd* were co-transformed into W3110 recipient cells. After 2 h of recovery at 32 °C in 1 mL of super optimal broth with catabolite repression (SOC) medium containing 20 g/L peptone, 5 g/L yeast extract, 0.5 g/L NaCl, 25 μmol/L KCl, 0.2 mmol/L MgCl_2_ and 0.4 mmol/L glucose, the cells were spread on Luria broth (LB) agar plates supplemented with spectinomycin and ampicillin and incubated at 32 °C for 12 h. Colony PCR screening was performed using primers *cdd*-QC-1 and *cdd*-QC-4, and the positive clones were subsequently verified by sequencing (GENEWIZ, Suzhou, China). The verified strains were then inoculated into LB broth supplemented with 10 mmol/L l-arabinose and incubated overnight at 32 °C to induce shutdown of the λ Red recombination system, thereby enabling the elimination of pGRB-*cdd*. After pGRB-*cdd* curing, the cultures were transferred to antibiotic-free LB broth and incubated overnight at 42 °C to promote loss of the pREDCas9 plasmid, which contains a temperature-sensitive origin of replication. The resulting cultures were serially diluted and plated onto antibiotic-free LB agar. Twenty-four single colonies were randomly selected and screened by colony PCR using plasmid-specific primers, and complete removal of pGRB-*cdd* and pREDCas9 was further confirmed by the inability of the isolates to grow on LB agar containing ampicillin and spectinomycin. Finally, each engineered strain was serially passaged for 10 generations under non-selective conditions, and 3–5 verified single colonies were selected for subsequent shake-flask fermentation and further engineering.

### 2.4 Fermentation experiments

#### 2.4.1 Shake-flask fermentation

The slant medium contained 2.0 g/L glucose, 10.0 g/L peptone, 5.0 g/L yeast extract, 2.5 g/L NaCl, 1.0 g/L KH_2_PO_4_, 0.2 g/L MgSO_4_ and 25 g/L agar, with water as the diluent; the pH was adjusted to 7.0-7.2. The seed medium contained 40 g/L glucose, 8 g/L yeast extract, 2 g/L peptone, 2 g/L (NH_4_)_2_SO_4_, 1 g/L MgSO_4_·7H_2_O, 3 g/L KH_2_PO_4_, 0.3 g/L methionine, 1 mg/L VB_2_, 2 mg/L each of VB_1_, VB_3_, VB_5_ and VB_12_, 1 mg/L biotin and 1 mL/L trace element solution. The fermentation medium contained 10 g/L glucose, 8 g/L yeast extract, 5 g/L glutamate, 5 g/L citrate, 3 g/L (NH_4_)_2_SO_4_, 2.5 g/L MgSO_4_·7H_2_O, 6 g/L KH_2_PO_4_, 0.5 g/L methionine, 40 mg/L FeSO_4_·7H_2_O, 10 mg/L MnSO_4_, 1.5 mg/L VB_2_, 1 mg/L each of VB_1_, VB_3_, VB_5_ and VB_12_, 0.5 mg/L biotin, 1 mL/L trace element solution and 8 mg/L phenol red; the pH was approximately 6.9. Cells from the cryopreserved stock were inoculated into liquid LB medium and activated at 36 °C for 12 h. Seed culture (2 mL) was then transferred to a 500 mL shake flask containing 30 mL of fermentation medium and incubated at 36 °C and 220 rpm for 24 h. Aqueous ammonia (25%, v/v) was added to control the fermentation pH according to the color change of phenol red. After the glucose in the initial broth was depleted, sterilized glucose solution (60%, w/v) was added as the carbon source for cell growth and product formation.

#### 2.4.2 Fed-batch fermentation in a 5 L bioreactor

Cells from the cryopreserved stock were inoculated onto the slant medium and activated at 36 °C for 12 h. A suitable number of activated cells was then added to a 5 L bioreactor (Shanghai Baoxing, China) containing 2 L of seed medium, and cultivation was carried out at 36 °C, with a pH range of 6.9 ± 0.2 and dissolved oxygen levels of 35%–45%. When optical density at 600 nm (OD_600_) reached 20, seed culture was transferred at a ratio of 20% (v/v) to a 5 L bioreactor containing 2 L of fermentation medium and incubated at 36 °C. Aqueous ammonia (25%, v/v) was added as needed to maintain a pH of 6.9 ± 0.2 during fermentation, and the agitation speed was adjusted to keep the dissolved oxygen concentration at 35%–45%. Sterile glucose solution (80%, w/v) was added as a concentrated carbon source to maintain the residual glucose concentration at or below 1 g/L. The seed and fermentation media used in the bioreactor were the same as those used in shake flasks, except that phenol red was omitted.

### 2.5 Analytical methods

Cell growth was measured by determining the absorbance at 600 nm using an ultraviolet-visible (UV-Vis) spectrophotometer (UV1800, Shanghai Jingke Technology Instrument Co., Ltd., Shanghai, China). An SBA-40E biosensor analyzer (Shandong Academy of Sciences, Shandong, China) was used to measure the glucose concentration. High-performance liquid chromatography (HPLC) on an LC-100 system equipped with a UV100 Plus ultraviolet detector was used to quantify the titers of cytidine and its byproducts. The analysis used an Agilent C18 column (250 mm × 4.6 mm, 5 μm), a mobile phase of phosphate buffer prepared from 1.884 g disodium hydrogen phosphate and 0.726 g sodium dihydrogen phosphate in 1000 mL deionized water mixed with acetonitrile (95:5, v/v), a column temperature of 30 °C, a flow rate of 1 mL/min, a detection wavelength of 260 nm and an injection volume of 20 μL.

Cells obtained after shake-flask fermentation were centrifuged at 4 °C and washed twice with phosphate-buffered saline (PBS) for ATP and NADH/NAD^+^ ratio assays. An enhanced firefly luciferase-based ATP assay kit (Beyotime, China) was used to measure the intracellular ATP level, and the protein concentration and luminescence of each sample were recorded on a microplate reader (BioTek, Winooski, VT, USA). The intracellular NADH/NAD^+^ ratio was measured using a coenzyme I NAD(H) assay kit (Solarbio, China). For the reporter assay, an appropriate volume of broth was collected and diluted 1:5 in fresh LB medium containing the corresponding antibiotic and inducer; Green fluorescent protein (GFP) abundance was then measured on the same microplate reader at an excitation wavelength of 488 ± 10 nm and an emission wavelength of 511 ± 10 nm, and all fluorescence values were normalized to the cell density determined by absorbance at 600 nm.

Cells from 1 mL of shake-flask culture were harvested at 4 °C and washed once with pre-chilled PBS. Cell pellets were extracted with 500 μL of pre-chilled 0.5 M perchloric acid, vortexed for 30–60 s, incubated on ice for 10 min, briefly sonicated, and kept on ice for an additional 5–10 min. After centrifugation at 12000 rpm for 10 min at 4 °C, the supernatant was neutralized to pH 7.0 with pre-chilled 2 M potassium carbonate. Following incubation on ice for 15–20 min, samples were centrifuged at 12000 rpm for 10 min at 4 °C to remove potassium perchlorate precipitates. The supernatant was transferred to a new tube and centrifuged again at 12000 rpm for 5 min at 4 °C. The final supernatant was filtered through a 0.22 μm hydrophilic membrane and analyzed by HPLC for intracellular UMP, UTP, CMP, and CTP.

### 2.6 Statistical analysis

Data are presented as the mean ± standard deviation of three independent experiments. One-way analysis of variance (ANOVA) was used to assess significant differences among groups, followed by Dunnett's multiple-comparison test. Statistical significance was defined as P < 0.05, and high statistical significance was defined as P < 0.01.

## 3 Results and discussion

### 3.1 Systematic attenuation of cytidine consumption pathways

Cytidine in wild-type *E. coli* is quickly broken down by several competing pathways, so it does not accumulate as a stable end product. Systematic attenuation of the catabolism of cytidine and of its direct precursor CMP is thus required for the accumulation of cytidine [24]. Cytidine degradation in *E. coli* mainly follows three pathways [25](Fig. 2A): cytidine deaminase (encoded by *cdd*) deaminates cytidine to uridine; the pyrimidine-specific nucleoside hydrolases (encoded by *rihA*, *rihB* and *rihC*) work together with the pyrimidine/purine nucleoside phosphorylase (encoded by *yaiE*) to break down cytidine into cytosine; and uridine-cytidine kinase (encoded by *udk*) phosphorylates cytidine to CMP. At the same time, CMP is directly upstream of cytidine and is divided into two competing pathways: hydrolysis of CMP to cytosine by pyrimidine/purine nucleotide 5′-monophosphate nucleosidase (encoded by *ygdH*), and further phosphorylation of CMP to CDP by cytidylate kinase (encoded by *cmk*), both of which reduce the supply efficiency of the precursor for cytidine synthesis.

**Fig. 2:**
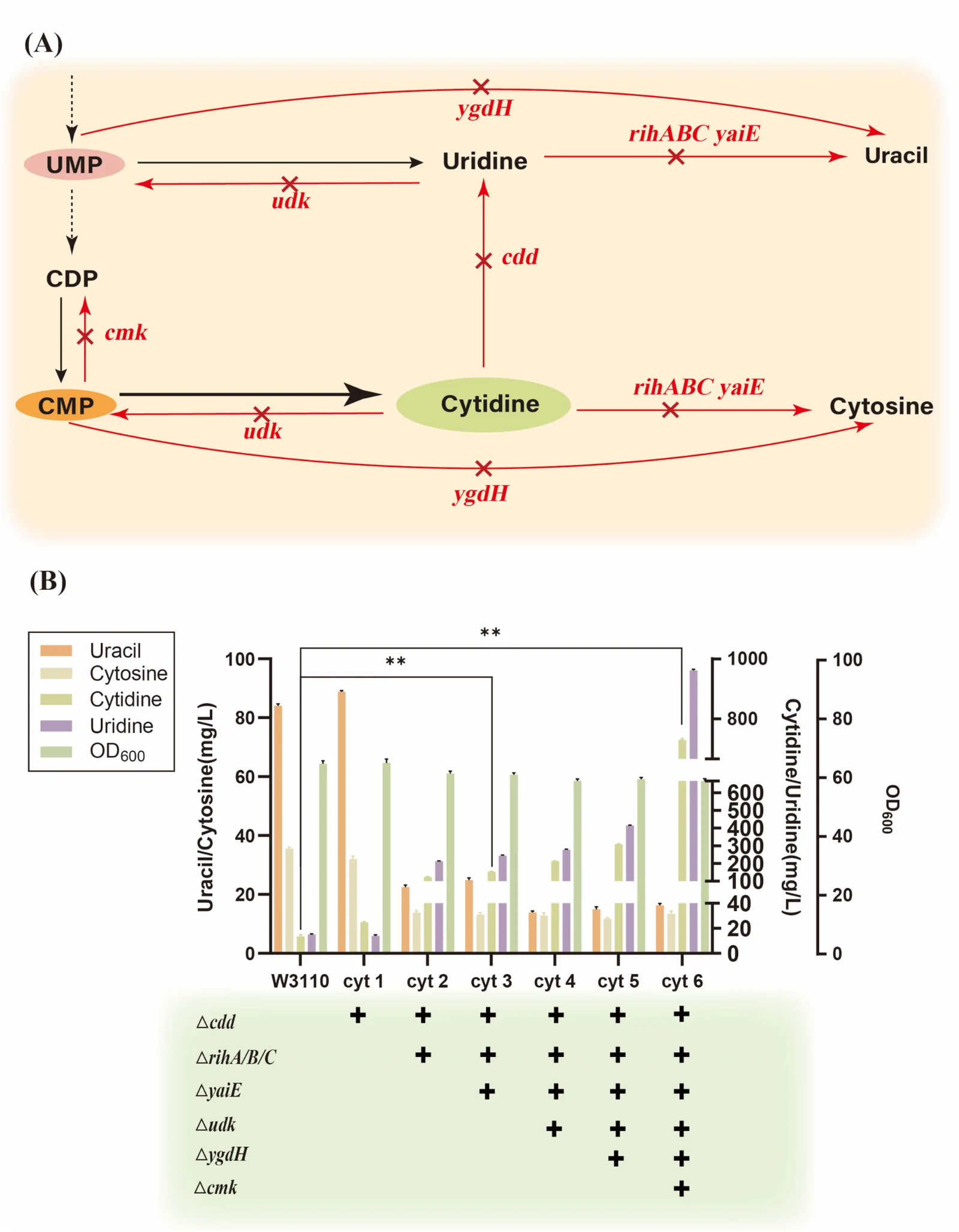
Improvement of strain performance by systematically attenuating cytidine catabolism. (A) Schematic representation of pathways competing for cytidine and CMP in *E. coli.* Red crosses indicate gene deletions. (B) Effects of stepwise deletion of *cdd*, *rihA/rihB/rihC*, *yaiE*, *udk*, *ygdH*, and *cmk* on cytidine, uridine, uracil, and cytosine accumulation, as well as cell growth. Data are presented as the mean ± standard deviation from three biological replicates. *P < 0.05; **P < 0.01.

To weaken these competing bypasses systematically, *cdd*, *rihA*/*rihB*/*rihC*, *yaiE*, *udk*, *ygdH* and *cmk* were sequentially deleted to obtain a series of progressively engineered deletion strains designated cyt 1 to cyt 6. Shake-flask fermentation (Fig. 2B) showed that the cytidine titer of the engineered strains increased stepwise as the deletion modules accumulated. Strain cyt 3, in which *cdd*, *rihA*/*rihB*/*rihC* and *yaiE* were deleted, was the first to accumulate cytidine strongly, reaching 153.43 mg/L, an increase of 1042.44% over wild-type W3110 (13.43 mg/L). Strain cyt 6 finally reached a cytidine titer of 730.20 mg/L, corresponding to an increase of 5337.08% compared with W3110; thus, targeted attenuation of cytidine degradation combined with suppression of CMP consumption significantly enhanced cytidine retention and accumulation.

Notably, the titer of the byproduct uridine also increased with the accumulation of deletion modules and reached 961.40 mg/L in the cyt 6 broth. These results indicated that a significant proportion of the metabolic flux was still directed toward the uridine branch; therefore, attenuation of cytidine degradation alone was insufficient for effective and selective cytidine accumulation. Because CMP is derived from UMP, a necessary intermediate in uridine synthesis, increasing the flux from UMP to CMP and then to cytidine could divert the competing branch and favor cytidine synthesis, which guided the following engineering strategy.

### 3.2 Reinforcement of the UMP-to-cytidine biosynthetic pathway

#### 3.2.1 Alleviation of feedback inhibition and enhancement of UMP-to-CMP conversion

The conversion of UMP to CMP in *E. coli* involves four consecutive enzymatic reactions (Fig. 3A). The rate-limiting enzymes of this pathway are CTP synthetase (encoded by *pyrG*) and UMP kinase (encoded by *pyrH*), both of which are feedback-inhibited by CTP and UTP, respectively. PyrH ^D93A^ and PyrG ^D160E, E162A, E168K^_cgl_ have been shown to significantly reduce feedback inhibition by UTP and CTP [26,27]. Additionally, nucleoside triphosphate pyrophosphohydrolase NudG degrades CTP to CMP, thereby reducing excess intracellular CTP and providing the direct precursor CMP for cytidine synthesis while relieving feedback inhibition. Based on this, we used cyt 6 as the parental strain and added different combinations of enzymatic elements, including wild-type the *pyrH* from *E. coli*, the *pyrH* ^D93A^ mutant from *E. coli*, the *pyrG* variant from *C. glutamicum*, the *pyrG* ^D160E, E162A, E168K^_cgl_ multi-site mutant from *C. glutamicum*, and *nudG* from *E. coli*, to construct engineered strains cyt 9-1 to cyt 9-4 (Fig. 3B). Shake-flask fermentation results (Fig. 3C) showed that the cytidine titers of strains cyt 6 to cyt 9-4 were 0.73 g/L, 1.44 g/L, 1.94 g/L, 1.87 g/L and 2.36 g/L, and the corresponding uridine titers were 0.92 g/L, 2.35 g/L, 2.14 g/L, 1.58 g/L and 1.32 g/L, respectively. The combination of *pyrH* ^D93A^, *pyrG* ^D160E, E162A, E168K^_cgl_ and *nudG* showed the best effect, increasing the cytidine titer by 223.28% compared with cyt 6 while maintaining a lower uridine byproduct level than the other combinations. These results indicated that this gene combination increased the metabolic flux of the cytidine synthesis pathway and promoted the accumulation of cytosine nucleotides.

**Fig. 3:**
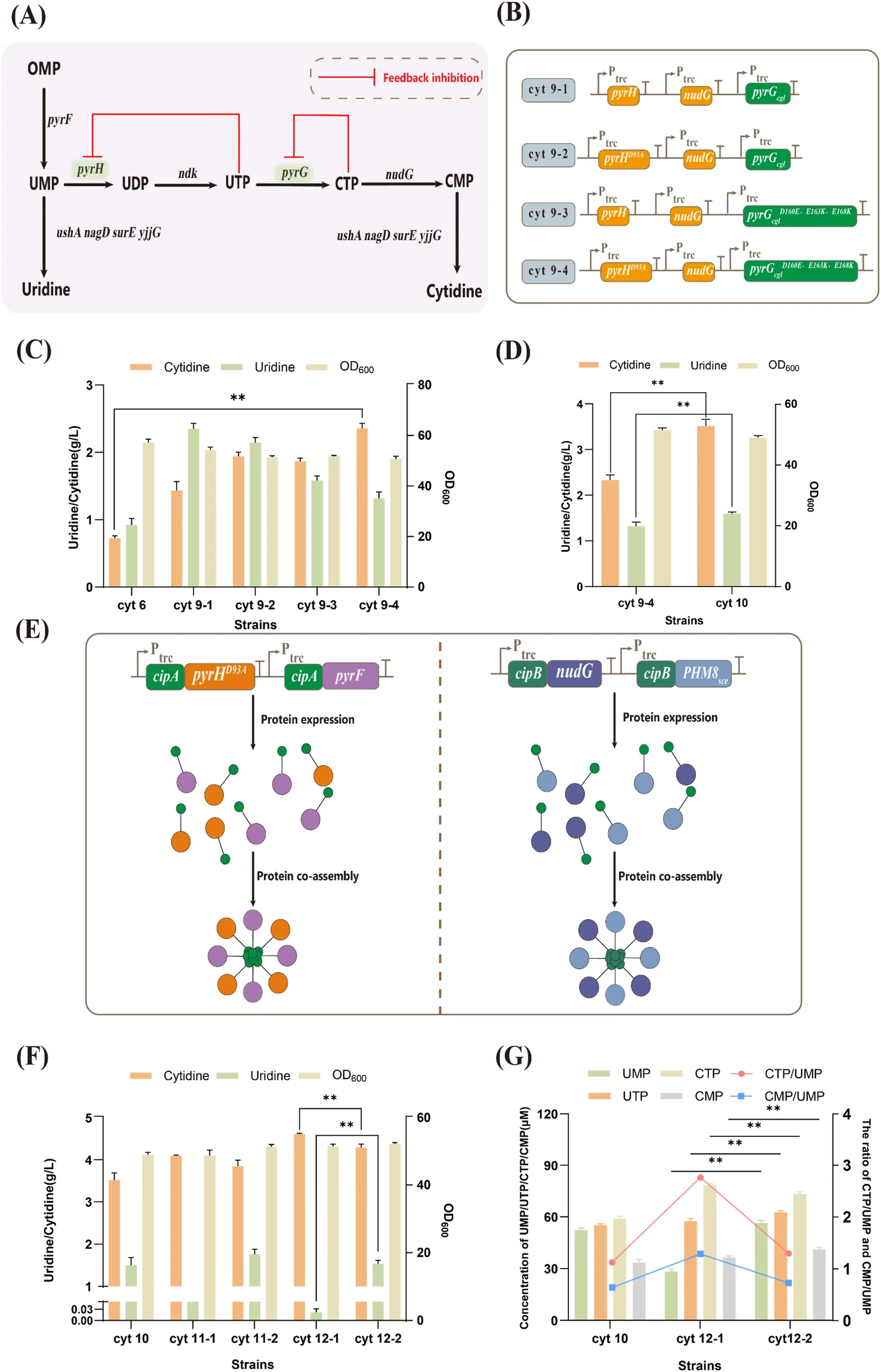
Improvement of strain performance by strengthening the UMP-to-cytidine pathway and applying multienzyme-complex channeling. (A) Schematic representation of the metabolic pathway converting UMP to cytidine. Red lines indicate feedback inhibition. (B) Schematic representation of engineered strains constructed with different combinations of enzymatic elements. (C) Effects of different enzymatic-element combinations on cytidine titer, uridine by-product accumulation, and cell growth. (D) Effect of introducing *PHM8*_sce_ from *S. cerevisiae* on cytidine titer and cell growth. (E) Schematic representation of the multienzyme-complex channeling strategy adopted in this study. (F) Comparison of cytidine titer, uridine byproduct accumulation, and cell growth between scaffold and non-scaffold strains in shake-flask fermentation. (G) Intracellular UMP, UTP, CTP, and CMP levels, and the CTP/UMP and CMP/UMP ratios, in cyt 10, cyt 12-1, and cyt 12-2. Data are presented as the mean ± standard deviation from three biological replicates. *P < 0.05; **P < 0.01.

#### 3.2.2 Enhancement of CMP-to-cytidine conversion by introducing a CMP-preferential 5′-nucleotidase

In the competition for cytidine and uridine, uridine is generally more likely to accumulate. Therefore, a certain amount of UMP can accumulate in the cell relatively easily due to the multi-step reaction process of UMP to CMP and strict feedback regulation, and this excess UMP will then be further utilized by endogenous 5′-nucleotidases [28]. In addition, the endogenous 5′-nucleotidases of *E. coli* have low substrate specificity and show catalytic activity toward both CMP and UMP with similar catalytic efficiency [29]. Endogenous 5′-nucleotidase with low substrate specificity would lead to a diversion of UMP into uridine; thus, the metabolic flux cannot be directed toward cytidine.

Therefore, a high-affinity 5′-nucleotidase for CMP was selected to improve cytidine synthesis efficiency and reduce uridine byproduct accumulation. Phosphate metabolism protein 8, a 5′-nucleotidase from *S. cerevisiae* (encoded by *PHM8*_*sce*_), has been reported to have a significantly higher substrate affinity for CMP and was therefore considered suitable for increasing cytidine synthesis [30]. We introduced codon-optimized *PHM8*_*sce*_ at the *ychG* locus of the cyt 9-4 chromosome under the control of the strong trc promoter to construct cyt 10. Shake-flask fermentation results (Fig. 3D) showed that the cytidine titer of cyt 10 increased by 51.07% compared with cyt 9-4, from 2.33 g/L to 3.52 g/L, whereas uridine production increased by only 21.21%, from 1.32 g/L to 1.60 g/L. These results indicated that heterologous expression of *PHM8*_sce_ preferentially promoted CMP-to-cytidine conversion over UMP-to-uridine conversion in vivo, which was consistent with its reported preference for CMP and effectively promoted cytidine accumulation.

#### 3.2.3 Scaffold-mediated enzyme clustering enhances flux channeling toward cytidine

Although the multi-level metabolic engineering modifications in the previous stage significantly increased cytidine production, the fermentation broth still showed a relatively high level of uridine accumulation at 1.60 g/L; thus, part of the metabolic flux was still diverted to the competing byproduct pathway. This may occur because the formation of both cytidine and uridine depends on 5′-nucleotidase-mediated dephosphorylation reactions. Nucleotide intermediates, such as UMP and CMP, are not spatially restricted in the cytoplasm and are diffusely distributed. Because UMP is located at an upstream metabolic node in the cytidine synthesis network, it is more readily captured by 5′-nucleotidases during diffusion and converted to uridine; thus, metabolic flux that should be directed toward cytidine synthesis is dispersed and lost [31,32]. To further improve metabolic partitioning and reduce uridine and other byproducts, we employed protein-scaffold channeling engineering to promote the assembly of essential enzymes, potentially reduce diffusion loss of intermediates, and direct metabolic flux toward the target product.

Based on these findings, a new channel structure using CipA/CipB scaffold proteins can enhance the collaboration of multiple enzymes and reduce byproducts. For example, the CipA self-assembling scaffold system has been successfully employed in *E. coli* to promote electron transfer between cytochrome P450 and its cognate reductase by achieving close spatial arrangements of the enzymes [33,34]. In addition, both CipA and CipB scaffold proteins can be expressed in a soluble state in *E. coli* without the need for external cofactors to mediate protein self-assembly, and they have shown good results in supporting the construction of multi-enzyme complexes and achieving significant reduction of byproducts [35,36].

On this basis, we constructed a CipA/CipB scaffold-mediated multi-enzyme assembly system on the cyt 10 chromosome (Fig. 3E). First, Ptrc-*cipA*-*pyrF* was introduced at the *ygaY* locus and Ptrc-*cipA*-*pyrH* at the *ycdN* locus to obtain the intermediate strain cyt 11-1. Next, based on cyt 11-1, Ptrc-*cipB*-*nudG* and Ptrc-*cipB*-*PHM8*_*sce*_ were added at the *ylbE* and *mbhA* loci, respectively, to obtain cyt 12-1. At the same time, cyt 10 was used as the parental strain, and the same genomic integration sites and promoter strengths were used to construct the control strains cyt 11-2 and cyt 12-2 without CipA/CipB scaffold proteins. Shake-flask fermentation results (Fig. 3F) showed that the multienzyme-complex strain cyt 12-1 reached a cytidine titer of 4.61 g/L, representing a 7.46% increase over the control strain cyt 12-2 (4.29 g/L). Meanwhile, uridine accumulation decreased from 1.54 g/L in cyt 12-2 to 0.02 g/L in cyt 12-1, corresponding to a 98.70% reduction. The two strains exhibited comparable OD_600_ values, suggesting that scaffold-mediated enzyme clustering did not impose a substantial growth burden.

To further examine pyrimidine flux redistribution, we quantified intracellular UMP, UTP, CTP, and CMP in cyt 10, cyt 12-1 and cyt 12-2 (Fig. 3G). Compared with cyt 12-2, cyt 12-1 showed a 49.84% decrease in UMP (from 56.50 to 28.34 μM), a 6.95% increase in CTP (from 73.27 to 78.36 μM), an 11.37% decrease in CMP (from 41.17 to 36.49 μM), and a 7.86% decrease in UTP (from 62.50 to 57.59 μM). Accordingly, the CTP/UMP and CMP/UMP ratios increased by 113.08% (1.30 to 2.77) and 76.71% (0.73 to 1.29), respectively. These changes indicate that CipA/CipB-mediated enzyme assembly altered intracellular pyrimidine nucleotide levels, most notably by reducing UMP accumulation and increasing the relative abundance of CTP. This effect may result from the spatial colocalization of PyrF, PyrH, NudG, and PHM8_sce_, which could shorten the diffusion distance of UMP- and CMP-related intermediates and facilitate their sequential utilization. Together with the 98.70% decrease in extracellular uridine, these data suggest that scaffold-mediated local flux coordination may reduce UMP diversion to uridine formation and thereby favor cytidine biosynthesis.

Notably, the strong decrease in uridine was not matched by a proportional increase in extracellular cytidine, indicating that only part of the redirected flux was recovered as product. The remaining flux may have been allocated to nucleotide homeostasis, salvage, biomass formation, transport, or other unmeasured processes.

Overall, the changes in intracellular pyrimidine nucleotide levels and extracellular product distribution suggest that CipA/CipB-mediated enzyme assembly may contribute to improved pyrimidine flux partitioning, reduced uridine byproduct formation, and enhanced cytidine production without an apparent growth penalty.

### 3.3 Reconstruction of the de novo pyrimidine nucleotide biosynthetic pathway

By blocking cytidine degradation, increasing the conversion rate from UMP to cytidine, and forming a multi-enzyme complex, the cytidine-forming capacity of the host was enhanced to some extent, but the supply of pyrimidine precursors was not improved. We therefore next increased UMP biosynthesis to provide a continuous precursor supply for cytidine synthesis.

In *E. coli*, the essential genes for de novo UMP synthesis, namely *carAB*, *pyrBI*, *pyrC*, *pyrD*, *pyrE* and *pyrF*, are located at different chromosomal positions, whereas the corresponding genes in *B. subtilis* are generally organized as a compact operon that has been used to construct *E. coli* strains overproducing pyrimidines [37,38]. To prevent the precursor carbamoyl phosphate from being diverted to arginine biosynthesis, *argF* was first deleted from the genome of cyt 12-1 to obtain cyt 13. Ptrc-*pyrB*-*pyrC*-*pyrAA*-*pyrAB*^E949^*-*pyrK*-*pyrD*-*pyrF*-*pyrE*, which was derived from *B. subtilis* F126 and is referred to as the pyr operon hereafter, was then introduced into the *yghX* locus of cyt 13 to obtain cyt 14 (Fig. 4A). Shake-flask fermentation (Fig. 4B) showed that the cytidine titer of cyt 14 was 99.17% higher than that of cyt 12, increasing from 4.83 g/L to 9.62 g/L. Introduction of the *B. subtilis* pyr operon reduced competition for carbamoyl phosphate and increased UMP availability, thereby promoting cytidine accumulation. A second copy of the pyr operon was then added to the *yghE* locus of cyt 14 to determine whether a higher gene dosage provided further benefits for cytidine synthesis; however, the double-copy strain cyt 15 showed only a 4.81% increase over cyt 14, from 9.77 g/L to 10.24 g/L (Fig. 4C). Because PyrE uses PRPP to convert orotate into orotidine 5′-monophosphate (OMP), which is subsequently decarboxylated to UMP by PyrF, PRPP availability was considered a plausible bottleneck after further amplification of the pyrimidine biosynthetic pathway. However, the limited additional improvement observed after introducing a second copy of the pyr operon may also be associated with constraints in other substrates required for de novo UMP biosynthesis, such as glutamine and aspartate. Therefore, subsequent engineering was designed to improve the supply of these relevant precursor pools and further support cytidine accumulation.

**Fig. 4:**
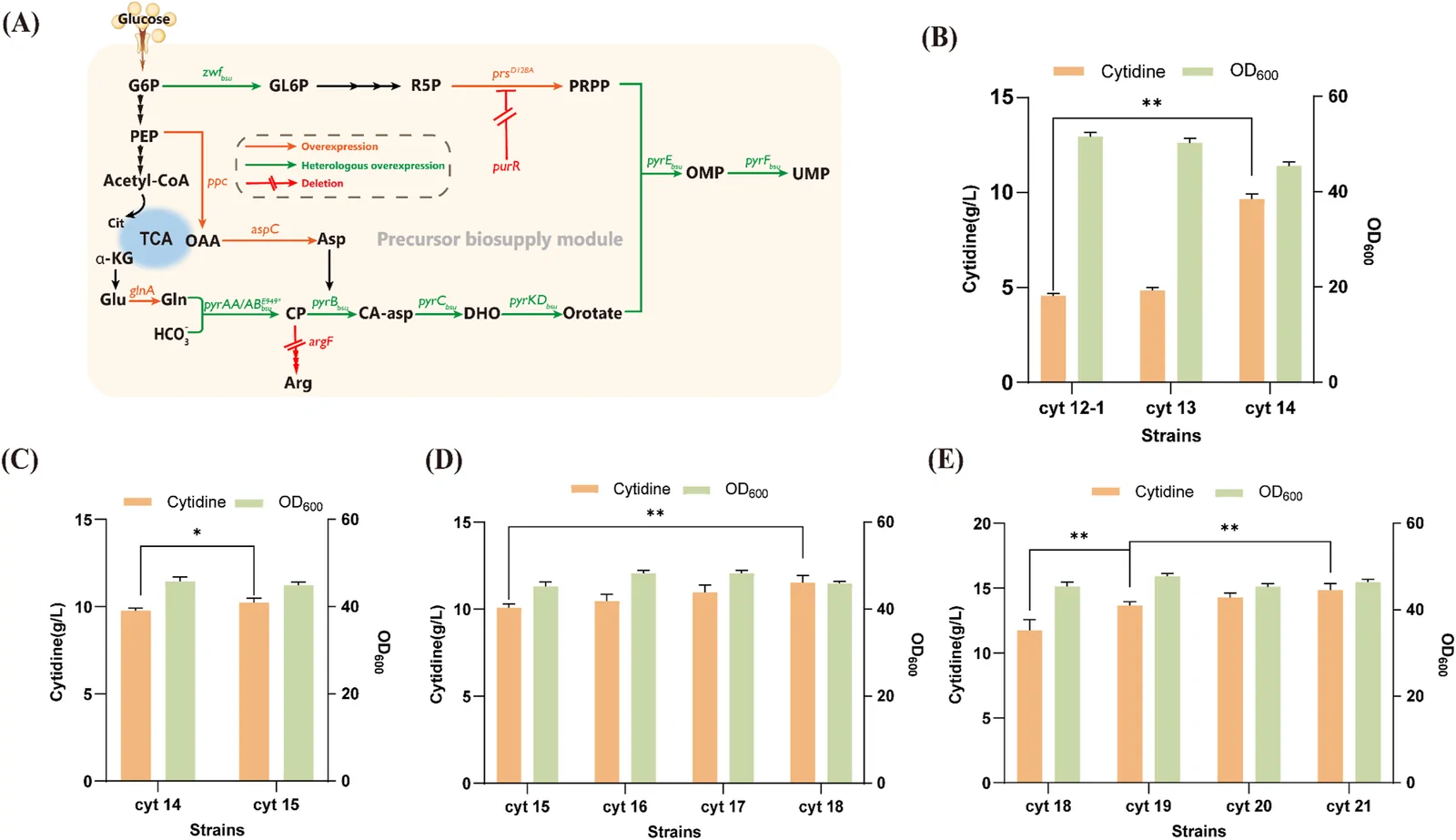
Improvement of strain performance by rewiring the de novo pyrimidine nucleotide biosynthesis pathway. (A) Schematic representation of the precursor-supply engineering strategy for reconstruction of the de novo pyrimidine nucleotide biosynthetic pathway. (B) Effects of *argF* deletion and introduction of the pyr operon from *B. subtilis* on cytidine titer and cell growth. (C) Effect of introducing a second copy of the pyr operon on cytidine titer and cell growth. (D) Effects of sequential *purR* deletion and introduction of *zwf*_bsu_ and *prs*^D128A^ on cytidine titer and cell growth. (E) Effects of sequential enhancement of glutamine- and aspartate-related genes on cytidine titer and cell growth. Data are presented as the mean ± standard deviation from three biological replicates. Data in different panels were obtained from independent shake-flask fermentation batches corresponding to successive construction rounds. The same strain may appear as the engineered strain in one panel and as the parental strain in a subsequent panel. *P < 0.05; **P < 0.01.

#### 3.3.1 Augmentation of PRPP supply

PRPP is produced through a series of enzymatic reactions in the pentose phosphate pathway (PPP) [39]. PRPP synthesis is carried out by PRPP synthetase (Prs, encoded by *prs*), and the expression and activity of Prs are negatively regulated by the HTH-type transcriptional repressor PurR (encoded by *purR*) [40]. To increase PRPP supply, *purR* was first deleted from the genome of cyt 15 to relieve transcriptional repression of the PRPP-related pathway. Ptrc-*zwf*_bsu_ was then attached to the *yciQ* locus to increase the metabolic flux of the oxidative branch of the PPP, and Ptrc-*prs*^D128A^ was introduced into the *gapC* locus to create a feedback-resistant Prs variant that alleviates inhibition of PRPP synthetase by downstream metabolites and enhances PRPP synthesis. Strains cyt 16, cyt 17 and cyt 18 were constructed in this order (Fig. 4A). Shake-flask fermentation (Fig. 4D) showed that the cytidine titer increased from 10.10 g/L in cyt 15 to 11.53 g/L in cyt 18, representing a 14.16% improvement; thus, relieving PurR-mediated transcriptional repression and strengthening PRPP biosynthesis alleviated PRPP limitation and further promoted cytidine accumulation.

#### 3.3.2 Co-optimization of glutamine and aspartate supply for de novo UMP synthesis

De novo UMP synthesis also requires a continuous supply of nitrogen-containing precursors, such as glutamine and aspartate. Glutamine is used in several amino group transfer reactions and serves as the primary nitrogen source for pyrimidine nucleotide synthesis [41]; aspartate is directly incorporated into the pyrimidine ring and is therefore required for UMP production [42]. Increasing the supply of PRPP or UMP alone is therefore insufficient to resolve precursor limitation, and co-optimization of upstream nitrogen metabolism and carbon skeleton supply is required. On this basis, Ptrc-*glnA* was inserted into the rph locus of cyt 18 to boost glutamine supply, and Ptrc-*ppc* and Ptrc-*aspC* were subsequently integrated at the *yjiT* and *yjiK* loci, respectively, to improve aspartate formation (Fig. 4A); thus, strains cyt 19, cyt 20 and cyt 21 were obtained. Shake-flask fermentation (Fig. 4E) showed that increased glutamine supply reinforced cytidine production in cyt 19 by 16.34%, from 11.75 g/L to 13.67 g/L, and additional aspartate supply further raised the titer of cyt 21 to 14.86 g/L, an increase of 8.71% over cyt 19. Strengthening the supply of both the nitrogen donor and the pyrimidine-ring precursor increased de novo flux toward UMP and provided a more abundant substrate pool for cytidine biosynthesis.

### 3.4 Dynamic reinforcement of energy and redox cofactor metabolism

#### 3.4.1 Identification of ATP limitation and validation of energy supplementation

To further improve cytidine biosynthesis, we systematically examined the cofactor and energy requirements of the key enzymatic reactions in the de novo cytidine biosynthetic pathway. De novo synthesis of one cytidine molecule in *E. coli* requires approximately six ATP molecules, and thus the intracellular ATP supply may be a bottleneck in cytidine synthesis. To determine whether enhancing intracellular ATP supply could promote cytidine accumulation, disodium ATP was added at different concentrations (0, 0.1, 0.2 and 0.3 g/L) to the shake-flask fermentation system of cyt 21, and the effects on cell growth and cytidine synthesis were investigated. The fermentation results showed that (Fig. 5A) addition of exogenous disodium ATP improved the cellular energy state. Cytidine synthesis performance and cell growth are shown in Fig. 5B; among the different supplementation concentrations, 0.2 g/L showed the most favorable effect, increasing the cytidine concentration by 13.84% (from 14.74 g/L to 16.78 g/L) and OD_600_ by 11.88% (from 46.63 to 52.17). Therefore, the positive response to ATP supplementation indicated that insufficient energy supply was at least one factor limiting cytidine synthesis.

**Fig. 5:**
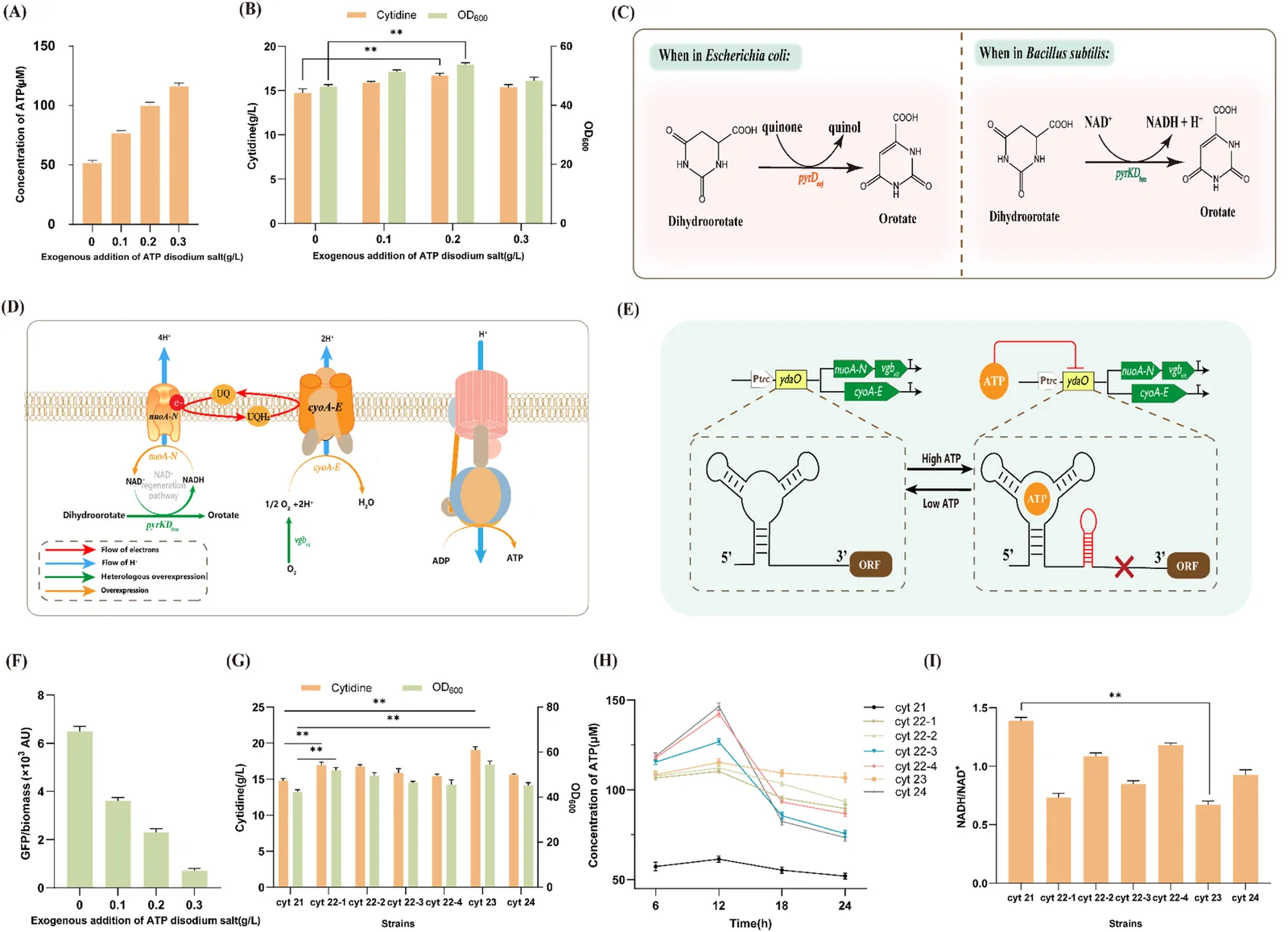
Improvement of strain performance by dynamically strengthening the respiratory chain to remodel energy and redox-cofactor metabolism. (A) Effects of supplementation with ATP disodium salt at different concentrations on intracellular ATP levels. (B) Effects of supplementation with ATP disodium salt at different concentrations on cytidine titer and cell growth. (C) Distinct cofactor requirements of *pyrD*_ecj_ and *pyrKD*_bsu_ for the oxidation of dihydroorotate to orotate. (D) Schematic representation of the respiratory-chain enhancement module. (E) Regulation of downstream gene expression by the *ydaO* motif in response to ATP. (F) Fluorescence intensity of *ydaO* motif-controlled GFP expression following exogenous supplementation with ATP disodium salt at different concentrations. (G) Effects of dynamic or static enhancement of different respiratory-chain modules on cytidine titer and cell growth. (H) Time-course profiles of intracellular ATP levels during shake-flask fermentation following dynamic or static enhancement of different respiratory-chain modules. (I) Effects of dynamic or static enhancement of different respiratory-chain modules on the intracellular NADH/NAD^+^ molar ratio. Data are presented as the mean ± standard deviation from three biological replicates. *P < 0.05; **P < 0.01.

#### 3.4.2 Dynamic respiratory-chain reinforcement coordinates ATP supply and NAD^+^ regeneration

Oxidative phosphorylation is a high-yield route for ATP generation under aerobic conditions. As the core of oxidative phosphorylation, the respiratory chain, also known as the electron transport chain (ETC), transfers electrons from NADH to terminal oxidases, pumps protons across the membrane to form a proton gradient, and thereby drives ATP production by ATP synthase; at the same time, NAD^+^ is regenerated through NADH oxidation to maintain cellular redox homeostasis [43,44]. The preceding results showed that cytidine synthesis is an energy-demanding process that requires ATP. In addition, the heterologous *pyrKD* introduced in this study was obtained from *B. subtilis* and requires NAD ^+^ to catalyze dihydroorotate oxidation to orotate, thereby consuming NAD^+^ and producing NADH (Fig. 5C); this may worsen cofactor-cycling stress in the host cell [45]. Therefore, strengthening respiratory-chain function may simultaneously improve ATP supply and NAD^+^ regeneration, thereby supporting cellular energy metabolism, redox homeostasis and efficient cytidine accumulation.

Based on the above reasoning, we selected an efficient respiratory-chain module for further host improvement (Fig. 5D). The module contained three parts: (i) NADH dehydrogenase I complex, encoded by *nuoA-N*, which transfers electrons from NADH to the ETC and pumps protons; (ii) cytochrome *bo*_3_ ubiquinol terminal oxidase, encoded by *cyoA-E*, which transfers electrons to oxygen and enhances the final oxidation capacity of the respiratory chain; and (iii) *Vitreoscilla* Hemoglobin (VHb), encoded by *vgb*_vit_, which efficiently binds oxygen and delivers it to the terminal region of the respiratory chain to increase cellular oxygen utilization [46].

Because increased respiratory-chain activity raises the intracellular ATP concentration, high ATP levels may promote cytidine synthesis but may also inhibit normal cell growth and product formation [47,48], an ATP-responsive regulatory strategy was considered. The *ydaO* motif is an ATP-responsive regulatory element. When intracellular ATP is sufficient, the *ydaO* motif can repress downstream gene expression; under low-ATP conditions, repression is lifted and the target module is expressed (Fig. 5E). This motif has been used to dynamically regulate genes related to ATP supply, maintain stable intracellular ATP levels, reduce metabolic burden and increase the production of products such as glutathione [49,50].

To confirm the regulatory function of the *ydaO* motif in *E. coli* W3110, a pTrc99a plasmid test system was constructed that contained the trc promoter, the *gfp* reporter gene and the *ydaO* motif as a regulatory element, and this system was introduced into W3110. Shake-flask fermentation was carried out for 24 h, GFP fluorescence intensity was measured at different concentrations of exogenous disodium ATP (0, 0.1, 0.2 and 0.3 g/L). As shown in Fig. 5F, GFP fluorescence intensity was only 0.7 × 10^3^ AU at high ATP concentration and reached 6.5 × 10^3^ AU at low ATP concentration. These experiments showed that the *ydaO* motif responded to ATP-related changes in the culture system and modulated downstream gene expression accordingly.Therefore, we introduced the *ydaO* motif to dynamically regulate the respiratory-chain enhancement module and balance cell growth with product synthesis.

Based on the above validation, Ptrc-*ydaO*-*nuoA-N*-*vgb*_vit_ and Ptrc-*ydaO*-*cyoA-E* were integrated into the *insB* and *molR* loci, respectively, using cyt 21 as the parental strain to construct cyt 22-1 and cyt 22-2. To distinguish the effect of dynamic regulation from that of respiratory-chain reinforcement itself, the corresponding static-expression control strains cyt 22-3 and cyt 22-4 were also constructed by integrating the same respiratory-chain modules without the *ydaO* motif into the same genomic loci. Shake-flask fermentation results (Fig. 5G) showed that both static and dynamically regulated respiratory-chain reinforcement were associated with higher cytidine titers and cell densities than those of cyt 21. Among these strains, cyt 22-1 showed the best observed performance, increasing the cytidine titer by 14.74% from 14.79 g/L to 16.97 g/L and increasing OD_600_ by 22.69% from 42.40 to 52.02. The corresponding static-expression strains also showed improved fermentation performance relative to cyt 21, but their overall performance was lower than that of the dynamically regulated strains.

Intracellular ATP profiling during shake-flask fermentation was consistent with the enhanced fermentation performance (Fig. 5H). Both static and dynamic respiratory-chain reinforcement increased ATP levels relative to cyt 21. However, the ATP time-course profiles differed between the two regulatory strategies. The dynamically regulated strains maintained relatively stable ATP levels throughout fermentation, whereas the static-expression strains showed more evident fluctuations or decreases at later time points. These results suggest that respiratory-chain reinforcement increased intracellular ATP availability, and that *ydaO*-mediated dynamic regulation may further contribute to ATP homeostasis during fermentation.

Next, Ptrc-*ydaO*-*cyoA-E* was introduced into the *molR* locus of cyt 22-1 to construct cyt 23, and the corresponding static-expression control strain was designated cyt 24. Shake-flask fermentation results (Fig. 5G and I) showed that both dynamic and static respiratory-chain reinforcement were again associated with higher cytidine titers than those of cyt 21, with cyt 23 showing the highest observed cytidine titer among these strains. Compared with cyt 21, cyt 23 increased the cytidine titer by 28.87% from 14.79 g/L to 19.06 g/L and increased OD_600_ by 28.70% from 42.40 to 54.57, while decreasing the NADH/NAD^+^ molar ratio by 51.80% from 1.39 to 0.67.

The ATP profile of cyt 23 also remained relatively stable during fermentation, whereas cyt 24 exhibited a less favorable ATP pattern (Fig. 5H). Overall, these results suggest that both static and dynamically regulated respiratory-chain reinforcement can improve cytidine production, but *ydaO*-mediated dynamic regulation may provide an additional benefit by maintaining a more stable intracellular energy state and a more balanced NADH/NAD^+^ ratio during fermentation, thereby supporting better coordination among energy metabolism, biomass formation and product synthesis.

### 3.5 Fed-batch fermentation for cytidine in 5-L bioreactors

To assess the production capability of the optimal engineered strain cyt 23, it was scaled up to a 5 L bioreactor for fed-batch cultivation. As shown in Fig. 6B, The logarithmic growth phase of the cells continued for 28 h and reached an OD_600_ of 142. Cytidine production entered a rapid accumulation phase after 14 h of fermentation, and the maximum instantaneous production rate of 3.12 g/(L·h) was achieved between 24 and 26 h. Strain cyt 23 finally accumulated 96.20 g/L cytidine after 50 h of fermentation, with a glucose conversion rate of 0.38 g/(g glucose) and an average productivity of 1.92 g/(L·h). To the best of our knowledge, these values represent the highest reported titer, glucose conversion rate and productivity for microbial cytidine biosynthesis to date, validating the effectiveness and practicality of the multi-level metabolic engineering strategy developed in this study.

**Fig. 6:**
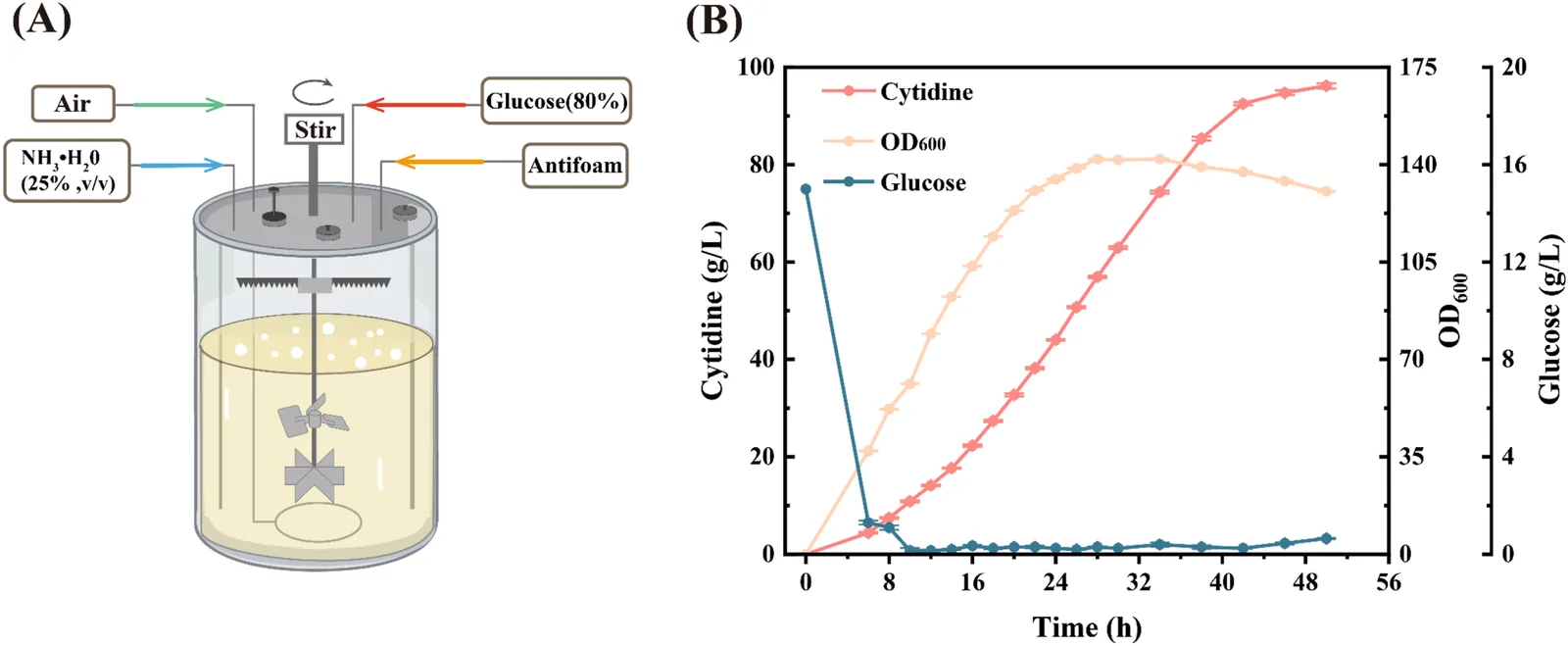
Validation of strain production performance during fed-batch fermentation. (A) Schematic representation of the 5-L bioreactor setup. (B) Conventional fed-batch fermentation of strain cyt 23 in a 5-L bioreactor.

## 4 Conclusions

This study established a plasmid-free *E. coli* platform for high-level cytidine production by coordinating pathway selectivity, precursor supply, byproduct control, and energy-redox metabolism. The plasmid-free chassis offers enhanced genetic stability, eliminates the need for antibiotic selection, and reduces the metabolic burden associated with plasmid maintenance, thereby providing a more robust and industrially relevant platform for large-scale fermentation. Cytidine accumulation was first enabled by blocking cytidine degradation, salvage, and competing CMP consumption. The flux from UMP to cytidine was then reinforced by relieving feedback inhibition, enhancing CMP formation, and introducing a CMP-preferential phosphatase. Importantly, CipA/CipB-mediated scaffold assembly spatially organized key enzymes and sharply decreased uridine accumulation, showing that local pathway channeling is an effective strategy for improving carbon allocation in pyrimidine nucleoside biosynthesis. Reconstruction of de novo UMP synthesis, enhancement of PRPP supply, and reinforcement of glutamine, oxaloacetate, and aspartate formation further expanded the precursor pool. In addition, *ydaO* motif-based dynamic regulation of respiratory-chain and oxygen-utilization modules improved the balance between ATP generation, NAD^+^ regeneration, and cell growth. The resulting strain cyt 23 reached 19.06 g/L cytidine in shake flasks and 96.20 g/L in 50 h in a 5 L fed-batch bioreactor, with a yield of 0.38 g/g glucose and productivity of 1.92 g/(L·h), representing the highest reported titer, yield and productivity for microbial cytidine production to date. These results demonstrate that efficient cytidine biosynthesis requires not only pathway amplification but also coordinated control of metabolic flux, enzyme organization, precursor availability, and cellular energy state. The strategy developed here offers a useful framework for constructing industrial microbial platforms for cytidine and related nucleoside products.Because the engineering modules are individually adjustable, this framework could potentially be adapted to other pyrimidine and purine nucleosides, as well as structurally modified derivatives, by redirecting pathway fluxes and tailoring precursor, cofactor, enzyme activity, and energy-redox requirements. Thus, the coordinated control of pathway selectivity, enzyme organization, precursor supply, byproduct formation, and cellular energy-redox state provides a transferable basis for developing microbial cell factories for diverse nucleoside products.

## CRediT authorship contribution statement

**Runze Zhao:** Writing – original draft, Methodology, Investigation, Data curation, Conceptualization. **Changgeng Li:** Writing – review & editing, Methodology, Investigation, Conceptualization. **Chaoye Xu:** Resources, Investigation. **Zhichao Chen:** Writing – review & editing. **Haibo Xiong:** Writing – original draft, Data curation. **Zichen Yu:** Visualization, Validation, Resources. **Tang'en Shi:** Visualization, Validation, Resources. **Lanxiao Li:** Visualization, Validation, Resources. **Meng Yuan:** Visualization, Validation, Resources. **Ling Ma:** Visualization, Validation, Resources. **Siyu Zhao:** Visualization, Validation, Resources. **Hongyu Deng:** Writing – review & editing, Supervision, Project administration, Funding acquisition. **Qingyang Xu:** Writing – review & editing, Supervision, Project administration, Funding acquisition.

## Declaration of generative AI and AI-assisted technologies in the writing process

During the preparation of this manuscript, the authors used ChatGPT to improve the clarity and readability of the language. The authors reviewed and revised the content thoroughly and accept full responsibility for the final version of the manuscript.

## Declaration of competing interests

The authors declare that they have no known competing financial interests or personal relationships that could have appeared to influence the work reported in this paper.

## ABBREVIATIONS

*E. coli*, *Escherichia coli*; CMP, cytidine monophosphate; UMP, uridine monophosphate; *B. subtilis*, *Bacillus subtilis*; PRPP, phosphoribosyl pyrophosphate; ATP, adenosine triphosphate; RNA, ribonucleic acid; ARTP, atmospheric and room-temperature plasma; UTP, uridine triphosphate; CTP, cytidine triphosphate; CRISPR/Cas9, Clustered Regularly Interspaced Short Palindromic Repeats/CRISPR-associated protein 9; DNA, deoxyribonucleic acid; *S. cerevisiae*, *Saccharomyces cerevisiae*; *C. glutamicum*, *Corynebacterium glutamicum*; PCR, polymerase chain reaction; dsDNA, double-stranded DNA; SOC, super optimal broth with catabolite repression; LB, Luria broth; UV-Vis, ultraviolet-visible; HPLC, high-performance liquid chromatography; PBS, phosphate-buffered saline; GFP, green fluorescent protein; OD_600_, optical density at 600 nm; ANOVA, analysis of variance; PPP, pentose phosphate pathway; ETC, electron transport chain, also known as respiratory chain; VHb, *Vitreoscilla* Hemoglobin.
