# Supplementary material for: Systems metabolic engineering of a plasmid-free Escherichia coli cell factory for high-level cytidine production
Source: Synth Syst Biotechnol. 2026 Sep 22;19:21–32. doi: 10.1016/j.synbio.2026.09.003 (PMC13654479; doi:10.1016/j.synbio.2026.09.003)
Supplement: Supplementary file 1 [file mmc1.docx]

**Systems metabolic engineering of a plasmid-free *Escherichia coli* cell factory for high-level cytidine production**

Runze Zhao^a,b,†^, Changgeng Li^a,b,†^, Chaoye Xu^a,b^, Zhichao Chen^a,b^, Haibo Xiong^a,b^, Zichen Yu^a,b^, Tang’en Shi^a,b^, Lanxiao Li^a,b^, Meng Yuan^a,b^, Ling Ma^a,b^, Siyu Zhao^a,b^, Hongyu Deng^c,*^, Qingyang Xu^a,b,*^

^a^ College of Biotechnology, Tianjin University of Science & Technology, Tianjin, 300457, P. R. China.

^b^ Key Laboratory of Industrial Fermentation Microbiology of the Ministry of Education, Tianjin University of Science & Technology, Tianjin, 300457, P. R. China.

^c^ State Key Laboratory of Green Papermaking and Resource Recycling, Qilu University of Technology (Shandong Academy of Science), Jinan, 250353, P. R. China

^†^ These authors contributed equally to this work.

^*^ Corresponding author: Qingyang Xu, Hongyu Deng

E-mail: xuqingyang@tust.edu

**Supplementary Table 1** Strains and plasmids used in this study

| Strains/Plasmids | Characteristics | Source |
| --- | --- | --- |
| **Strains** |  |  |
| *E.coli* DH5α | the cloning host | This lab |
| *E.coli* W3110 | wild type, starting strain | This lab |
| *B. subtilis* F126 | Donor of *pyrB*, *pyrC*, *pyrAA*, *pyrAB^E949*^*, *pyrK*, *pyrD*, *pyrF*, *pyrE, zwf* genes and *ydaO* motif | This lab |
| *C. glutamicum* | Donor of *pyrG* ^D160E, E162A, E168K^_cgl_ gene | This lab |
| cyt 1 | W3110, *Δcdd* | This study |
| cyt 2 | cyt 1, *ΔrihA/rihB/rihC* | This study |
| cyt 3 | cyt 2, *ΔyaiE* | This study |
| cyt 4 | cyt 3, *Δudk* | This study |
| cyt 5 | cyt 4, *ΔygdH* | This study |
| cyt 6 | cyt 5, *Δcmk* | This study |
| cyt 9-1 | cyt 6, *yeeL:*: *Ptrc-pyrH, yeeP:*: *Ptrc-nudG, yjgX:: Ptrc-pyrG_cgl_* | This study |
| cyt 9-2 | cyt 6, *yeeL:*: *Ptrc-pyrH^D93A^, yeeP:*: *Ptrc-nudG, yjgX:: Ptrc-pyrG_cgl_* | This study |
| cyt 9-3 | cyt 6, *yeeL:*: *Ptrc-pyrH, yeeP:*: *Ptrc-nudG, yjgX:: Ptrc-pyrG^D160E, E162A, E168K^_cgl_* | This study |
| cyt 9-4 | cyt 6, *yeeL:*: *Ptrc-pyrH^D93A^, yeeP:*: *Ptrc-nudG, yjgX:: Ptrc-pyrG^D160E, E162A, E168K^_cgl_* | This study |
| cyt 10 | cyt 9-4, *ychG:*: *Ptrc-PHM8_sce_* | This study |
| cyt 11-1 | cyt 10, *ygaY*:: *Ptrc-cipA-pyrF，ycdN*:*: Ptrc-cipA-pyrH^D93A^* | This study |
| cyt 11-2 | cyt 10, *ygaY*:: *Ptrc-pyrF，ycdN*:: *Ptrc-pyrH^D93A^* | This study |
| cyt 12-1 | cyt 11-1, *ylbE*:: *Ptrc-cipB-nudG, mbhA*:: *Ptrc-cipB-PHM8_sce_* | This study |
| cyt 12-2 | cyt 11-2, *ylbE*:: *Ptrc-nudG, mbhA*:: *Ptrc-PHM8_sce_* | This study |
| cyt 13 | cyt 12-1, *ΔargF* | This study |
| cyt 14 | cyt 13, *yghX*:: *Ptrc-pyrB-pyrC-pyrAA-pyrAB^E949*^-pyrK-pyrD-pyrF-pyrE (bsu)* | This study |
| cyt 15 | cyt 14, *yghE*:: *Ptrc-pyrB-pyrC-pyrAA-pyrAB^E949*^-pyrK-pyrD-pyrF-pyrE (bsu)* | This study |
| cyt 16 | cyt 15, *ΔpurR* | This study |
| cyt 17 | cyt 16, *yciQ*:: *Ptrc-zwf_bsu_* | This study |
| cyt 18 | cyt 17, *gapC*:: *Ptrc-prs^D128A^* | This study |
| cyt 19 | cyt 18, *rph*:: *Ptrc-glnA* | This study |
| cyt 20 | cyt 19, *yjiT*:: *Ptrc-ppc* | This study |
| cyt 21 | cyt 20, *yjiK*:: *Ptrc-aspc* | This study |
| cyt 22-1 | cyt 21, *insB*:: *Ptrc-ydaO-nuoA-N-vgb_vit_* | This study |
| cyt 22-2 | cyt 21, *molR*:: *Ptrc-ydaO-cyoA-E* | This study |
| cyt 22-3 | cyt 21, *insB*:: *Ptrc-nuoA-N-vgb_vit_* | This study |
| cyt 22-4 | cyt 21, *molR*:: *Ptrc-cyoA-E* | This study |
| cyt 23 | cyt 22-1, *molR*:: *Ptrc-ydaO-cyoA-E* | This study |
| cyt 24 | cyt 22-3, *molR*:: *Ptr-cyoA-E* | This study |
| **Plasmids** |  |  |
| pREDCas9 | Spe^r^，Cas9 and λ Red expression vector |  |
| pGRB | Amp^r^，gRNA expression vector |  |
| pTrc99a | Amp^r^ expression vector |  |

**Supplementary Table 2** Primers used in this study

| **Primers** | **Sequence (5’→ 3’)** |
| --- | --- |
| *cdd-*QC-1 | TTATCAGGAAGGTGCATTTAGTTCGCT |
| *cdd-*QC-2 | CCAGCGGAAGTAGTGCGAATG |
| *cdd-*QC-3 | GCAGAAAAAGCCGATGCGC |
| *cdd-*QC-4 | AGATATAAGGCGCGGTTTTCCAG |
| pGRB-*cdd*-S | AGTCCTAGGTATAATACTAGTTGCCTTTGGGCCGAAAGATCGTTTTAGAGCTAGAA |
| pGRB-*cdd*-A | TTCTAGCTCTAAAACGATCTTTCGGCCCAAAGGCAACTAGTATTATACCTAGGACT |
| *rihA-*QC-1 | GACTCGCCGAAAGACGCTT |
| *rihA-*QC-2 | CTCAACAGAGGTAAATAACTCCGGTCAGCATACGCAGAACATTGCGTA |
| *rihA-*QC-3 | TACGCAATGTTCTGCGTATGCTGACCGGAGTTATTTACCTCTGTTGAG |
| *rihA*-QC-4 | CGTCTTTCCAGACGGCAATTAAACT |
| pGRB-*rihA*-S | GCGCTATGGGGCTGGGTAACGTTTTAGAGCTAGAAATAGCAAGTTAA |
| pGRB-*rihA*-A | GTTACCCAGCCCCATAGCGCACTAGTATTATACCTAGGACTGAGC |
| *rihB-*QC-1 | ATGGAAAAGAGAAAAATTATTCTGGATTGTGATCC |
| *rihB-*QC-2 | AATCACGTCCGGGGTGCCGATATTTGAAAGCGGACCAACCG |
| *rihB-*QC-3 | CGGTTGGTCCGCTTTCAAATATCGGCACCCCGGACGTGATT |
| *rihB*-QC-4 | TTAATGGGTTTTGATGTAGCCGCG |
| pGRB-*rihB*-S | AATCCGTGAAATTGTGCTAAGTTTTAGAGCTAGAAATAGCAAGTTAA |
| pGRB-*rihB*-A | TTAGCACAATTTCACGGATTACTAGTATTATACCTAGGACTGAGC |
| *rihC-*QC-1 | ATGCGTTTACCTATCTTCCTCGATACTGA |
| *rihC-*QC-2 | CATCCAAACCGCACATGACGATCGGGCCGATGGCCAC |
| *rihC-*QC-3 | GTGGCCATCGGCCCGATCGTCATGTGCGGTTTGGATG |
| *rihC*-QC-4 | TTACGACGCCAGAGCCAG |
| pGRB-*rihC*-S | CAAGCCGTATATTCGCCGTCGTTTTAGAGCTAGAAATAGCAAGTTAA |
| pGRB-*rihC*-A | GACGGCGAATATACGGCTTGACTAGTATTATACCTAGGACTGAGC |
| *yaiE-*QC-1 | GATGTCATCATGCTGGATTGTTTAGGA |
| *yaiE-*QC-2 | CAAGGGGAAGGCGAGGAATTACAAAACTGGCCCTGTTTGGTAGTTG |
| *yaiE-*QC-3 | CAACTACCAAACAGGGCCAGTTTTGTAATTCCTCGCCTTCCCCTTG |
| *yaiE*-QC-4 | CACCATTCATCATCTGATTTATCTTTTATCGTAGC |
| pGRB-*yaiE*-S | GAAGAGATGACGGTAATCAGGTTTTAGAGCTAGAAATAGCAAGTTAA |
| pGRB-*yaiE*-A | CTGATTACCGTCATCTCTTCACTAGTATTATACCTAGGACTGAGC |
| *udk-*QC-1 | CGCAACTGCGTTTAACATTTTTTACCT |
| *udk-*QC-2 | CTGAACGGTACACAATTTATCAAGCTTATTCCTGATCAGTCATATATTTAGCGACCTGAT |
| *udk-*QC-3 | ATCAGGTCGCTAAATATATGACTGATCAGGAATAAGCTTGATAAATTGTGTACCGTTCAG |
| *udk*-QC-4 | GGCGTACATCCACCGTCG |
| pGRB-*udk*-S | TAACGAGCGTGGGCGTTCAAGTTTTAGAGCTAGAAATAGCAAGTTAA |
| pGRB-*udk*-A | TTGAACGCCCACGCTCGTTAACTAGTATTATACCTAGGACTGAGC |
| *ygdH-*QC*-1* | ATGGCACTTGCATTGATGACCA |
| *ygdH-*QC-2 | CCTGGCAACTTCATACGATGCTGGGTATAACGTGCGTAAACGCTCAAT |
| *ygdH-*QC-3 | ATTGAGCGTTTACGCACGTTATACCCAGCATCGTATGAAGTTGCCAGG |
| *ygdH-*QC-4 | GCGTGAGGGTGGTAAGAAACA |
| pGRB*-ygdH*-S | GCGAATTATTCGCGCTTTGCGTTTTAGAGCTAGAAATAGCAAGTTAA |
| pGRB*-ygdH*-A | GCAAAGCGCGAATAATTCGCACTAGTATTATACCTAGGACTGAGC |
| *cmk-*QC-1 | TTGTCATCTTTCGGGCTGGTC |
| *cmk-*QC-2 | GCCGGAACCAGTGGCGCTACCGCTCGGTTACGATAGTCCAGCAGATGCCATTGC |
| *cmk-*QC-3 | GAAGCGTTGCAATGGCATCTGCTGGACTATCGTAACCGAGCGGTAGC |
| *cmk-*QC*-4* | GCAACAACAACGCCACGAAC |
| pGRB*-cmk*-S | GATTGCCGATGGCCGCGACAGTTTTAGAGCTAGAAATAGCAAGTTAA |
| pGRB*-cmk*-A | TGTCGCGGCCATCGGCAATCACTAGTATTATACCTAGGACTGAGC |
| *yeeL-*U-S | GGCGGTGATGCCATAGAGTAAAAAATC |
| *yeeL*-U-A | TGTTATCCGCTCACAATTCCACACATTATACGAGCCGGATGATTAATTGTCAATGGCCATAGCATCGCCAATCT |
| *yeeL*-D-S | GCCTTTCGTTTTATCTGTTGTTTGTCGGTGAACGCTCTCCTGAGTAGGACAAATGGTGAAGATAAAGCCAGGGCATTAG |
| *yeeL*-D-A | CATTCCCTCTACAGAACTAGCCCTTC |
| PGRB-*yeeL*-S | TCTTTCCTGTTATTCGGGCGGTTTTAGAGCTAGAAATAGCAAGTTAA |
| PGRB-*yeeL*-A | CGCCCGAATAACAGGAAAGAACTAGTATTATACCTAGGACTGAGC |
| *pyrH*-trc-UP-S | TCGTATAATGTGTGGAATTGTGAGCGGATAACAATTTCACACAGGAAACAGACCATGGCTACCAATGCAAAACCC |
| *pyrH*-UP-A | GCAGCACGCATTGCCAGGCCGTTCATTACGGTCGC |
| *pyrH*-DW-S | CTGGCAATGCGTGCTGCACTGCACCGCGCCTATGT |
| *pyrH*-DW-trc-A | CACCGACAAACAACAGATAAAACGAAAGGCCCAGTCTTTCGACTGAGCCTTTCGTTTTATTTGTTATTCCGTGATTAAAGTCCCTTCT |
| *yjgX*-U-S | GGAAGTCAACGGGTTATGCG |
| *yjgX*-U-A | AATTGTTATCCGCTCACAATTCCACACATTATACGAGCCGGATGATTAATTGTCAAAAAATCACCACGAATACCAGAATC |
| *yjgX*-D-S | CTGGGCCTTTCGTTTTATCTGTTGTTTGTCGGTGAACGCTCTCCTGAGTAGGACAAATACAGTGTCTTCCCTGAGCCG |
| *yjgX*-D-A | GGCGAAGGATACCATCAAGC |
| PGRB-*yjgX*-S | TCGCGACCACCGTAACTGGCGTTTTAGAGCTAGAAATAGCAAGTTAA |
| PGRB-*yjgX*-A | GCCAGTTACGGTGGTCGCGAACTAGTATTATACCTAGGACTGAGC |
| *pyrG-cgl*-trc-UP-S | TCGTATAATGTGTGGAATTGTGAGCGGATAACAATTTCACACAGGAAACAGACCATGACCTCTAGTCGAAAAGTCCGT |
| *pyrG-cgl*-UP-A | TTAAGGAATGGCTGGGATGCAATCTCACCGACGGTGCCACCG |
| *pyrG-cgl*-DW-S | GAGATTGCATCCCAGCCATTCCTTAAAGCAGCTCGCCAGGTACG |
| *pyrG-cgl*-DW-trc-A | AACAACAGATAAAACGAAAGGCCCAGTCTTTCGACTGAGCCTTTCGTTTTATTTGCTAAGGGTGGACACGCAGCT |
| *yeeP*-U-S | GGTCAGGAGGTAACTTATCAGCGC |
| *yeeP*-U-A | ATTGTTATCCGCTCACAATTCCACACATTATACGAGCCGGATGATTAATTGTCAAATGGCAGGGCTCCGTTTTG |
| *yeeP*-D-S | CTTTCGTTTTATCTGTTGTTTGTCGGTGAACGCTCTCCTGAGTAGGACAAATCTGTTTTCGTTACTGTCTCTCTTGTCC |
| *yeeP*-D-A | CGATGTCAGCAGCCAGCAC |
| PGRB-*yeeP*-S | TGAACAGTTTACCGGTGCGGGTTTTAGAGCTAGAAATAGCAAGTTAA |
| PGRB-*yeeP*-A | CCGCACCGGTAAACTGTTCAACTAGTATTATACCTAGGACTGAGC |
| *nudG*-trc-S | TTGTGAGCGGATAACAATTTCACACAGGAAACAGACCATGAAAATGATTGAAGTTGTTGCCGC |
| *nudG*-trc-A | AGATAAAACGAAAGGCCCAGTCTTTCGACTGAGCCTTTCGTTTTATTTGCTAATCCGCTGGTCTGGCG |
| *ygaY*-U-S | CCTACAAACCACATCGCACATT |
| *ygaY*-U-A | AATTGTTATCCGCTCACAATTCCACACATTATACGAGCCGGATGATTAATTGTCAAACACCGAAGCAACCCAAAAG |
| *ygaY*-D-S | AAAGACTGGGCCTTTCGTTTTATCTGTTGTTTGTCGGTGAACGCTCTCCTGAGTAGGACAAATTTGCTTGCCGCTCCACC |
| *ygaY*-D-A | GGAGTAGGGCTTTCCATAGAGTGT |
| PGRB-*ygaY*-S | CTCAACTACCCACAGTTGTTGTTTTAGAGCTAGAAATAGCAAGTTAA |
| PGRB-*ygaY*-A | AACAACTGTGGGTAGTTGAGACTAGTATTATACCTAGGACTGAGC |
| *ycdN*-U-S | GATTTTGACGCCACCAACACC |
| *ycdN*-U-A | AATTGTTATCCGCTCACAATTCCACACATTATACGAGCCGGATGATTAATTGTCAACCAATCCACATCACACAATCCAT |
| *ycdN*-D-S | AAAGACTGGGCCTTTCGTTTTATCTGTTGTTTGTCGGTGAACGCTCTCCTGAGTAGGACAAATGAAGGGATTTTTGGCTATCAGGA |
| *ycdN*-D-A | CATATCGTATTCGCCAGGCTG |
| PGRB-*ycdN-S* | AGTCCTAGGTATAATACTAGTGCGGCATTTCAACAAGATGTGTTTTAGAGCTAGAA |
| PGRB-*ycdN*-A | TTCTAGCTCTAAAACACATCTTGTTGAAATGCCGCACTAGTATTATACCTAGGACT |
| *cipA*-trc-s | TCGTATAATGTGTGGAATTGTGAGCGGATAACAATTTCACACAGGAAACAGACCATGATTAACGATATGCATCCGAGCC |
| *cipA-pyrF*-A | GGAAGAAGATGAAGCAGTTAACGTCATCATGCTAATTTCCACGCAGTTAATATAATCG |
| *cipA-pyrF*-S | CGATTATATTAACTGCGTGGAAATTAGCATGATGACGTTAACTGCTTCATCTTCTTCC |
| *pyrF*-trc-A | AACAACAGATAAAACGAAAGGCCCAGTCTTTCGACTGAGCCTTTCGTTTTATTTGTCATGCACTCCGCTGTAAAGAGG |
| *pyrF*-trc-S | GTATAATGTGTGGAATTGTGAGCGGATAACAATTTCACACAGGAAACAGACCATGACGTTAACTGCTTCATCTTCTTCC |
| *cipA-pyrH*-A | GGGTTTTGCATTGGTAGCCATCATGCTAATTTCCACGCAGTTAATATAATCG |
| *cipA-pyrH*-S | CGATTATATTAACTGCGTGGAAATTAGCATGATGGCTACCAATGCAAAACCC |
| *ylbE*-U-S | ACCCAACCTTACGCAACCAGT |
| *ylbE*-U-A | GTTATCCGCTCACAATTCCACACATTATACGAGCCGGATGATTAATTGTCAATTGTTCGATAACCGCAGCATTGG |
| *ylbE*-D-S | AGACTGGGCCTTTCGTTTTATCTGTTGTTTGTCGGTGAACGCTCTCCTGAGTAGGACAAATCGCTGGCGTGCTTTGAAC |
| *ylbE*-D-A | GGCGTAACTCAGCAGGCA |
| PGRB-*ylbE*-S | ACACTGGCTGGATGTGCAACGTTTTAGAGCTAGAAATAGCAAGTTAA |
| PGRB-*ylbE*-A | GTTGCACATCCAGCCAGTGTACTAGTATTATACCTAGGACTGAGC |
| *mbhA-U-S* | GCCAGCACGAACATAATCCCG |
| *mbhA*-U-A | TGTTATCCGCTCACAATTCCACACATTATACGAGCCGGATGATTAATTGTCAACACGGTGGCAGGTTTTGG |
| *mbhA*-D-S | CTGGGCCTTTCGTTTTATCTGTTGTTTGTCGGTGAACGCTCTCCTGAGTAGGACAAATGACCAAAAGTGCGTCCGATACG |
| *mbhA*-D-A | CGGCGTAATCACAAACTGGC |
| PGRB-*mbhA* -S | GCGTGATGTGAATGAGAAAAGTTTTAGAGCTAGAAATAGCAAGTTAA |
| PGRB-*mbhA*-A | TTTTCTCATTCACATCACGCACTAGTATTATACCTAGGACTGAGC |
| *cipB*-trc-s | ATGTGTGGAATTGTGAGCGGATAACAATTTCACACAGGAAACAGACCATGATTATCAAGAAAGATATTCTGCTTAACGAAGAAC |
| *cipB-nudG*-A | GCGGCAACAACTTCAATCATTTTCATGATTTCCACACCCACAATGTATTTAATCTTTGTA |
| *cipB-nudG*-S | TACAAAGATTAAATACATTGTGGGTGTGGAAATCATGAAAATGATTGAAGTTGTTGCCGC |
| *cipB-PHM8*_sce_-A | GGTTTCTGTATATGGTTCTATAATCTTTTGCAATTGTCATGATTTCCACACCCACAATGTATTTAATCTTTGTA |
| *cipB-PHM8*_sce_-S | TACAAAGATTAAATACATTGTGGGTGTGGAAATCATGACAATTGCAAAAGATTATAGAACCATATACAGAAACC |
| *PHM8*_sce_-trc-A | CAGATAAAACGAAAGGCCCAGTCTTTCGACTGAGCCTTTCGTTTTATTTGTTATGATGATTGTACGTTGATTTGTTG |
| *PHM8*_sce_-trc-S | TGGAATTGTGAGCGGATAACAATTTCACACAGGAAACAGACCATGACAATTGCAAAAGATTATAGAACC |
| *argF-*QC-1 | CGGAAATGGACGAACAGTGG |
| *argF-*QC-2 | AACTCCTTCGCCATCTGCTTCTTACAAAGGTGGAGGCAAACC |
| *argF-*QC-3 | GGTTTGCCTCCACCTTTGTAAGAAGCAGATGGCGAAGGAGTT |
| *argF-*QC*-4* | TTAGCAGCGAGTGGAGGGTAC |
| pGRB*-argF*-S | AGTCCTAGGTATAATACTAGTCAGAGAAGTGAACTGTGCAGGTTTTAGAGCTAGAA |
| pGRB*-argF*-A | TTCTAGCTCTAAAACCTGCACAGTTCACTTCTCTGACTAGTATTATACCTAGGACT |
| *yghX-*U-S | GCGCAACGTAGAACAGGAATT |
| *yghX-*U-A | TGTGTGAAATTGTTATCCGCTCACAATTCCACACATTATACGAGCCGGATGATTAATTGTCAAGATTGAAGCGCCTTTACTACTCC |
| *yghX*-D-S | AAAGACTGGGCCTTTCGTTTTATCTGTTGTTTGTCGGTGAACGCTCTCCTGAGTAGGACAAATGTCATAGTAATCCAGCAACTCTTGTG |
| *yghX*-D-A | GAGCAGGTATTTACGTGAACCG |
| *yghX*-4#-S | ATGCACAGGAGACTTTCTGATGCGCTGGTTGATTTCTTCTAGGGTCATAGTAATCCAGCAACTGATTGAAGCGCCTTTACTACTCC |
| *yghX*-1#-S | CTGAACAACATCATATTTAAATGAACATAACTCAATTTGTAGGCTAGCATAACCCCTTGGGGCGTCATAGTAATCCAGCAACTCTTGTG |
| PGRB-4#-S | TGCGCTGGTTGATTTCTTCTGTTTTAGAGCTAGAAATAGCAAGTTAA |
| PGRB-4#-A | AGAAGAAATCAACCAGCGCAACTAGTATTATACCTAGGACTGAGC |
| PGRB-1#-S | ATGAACATAACTCAATTTGTGTTTTAGAGCTAGAAATAGCAAGTTAA |
| PGRB-1#-A | ACAAATTGAGTTATGTTCATACTAGTATTATACCTAGGACTGAGC |
| PGRB-*yghX*-S | TGTGGCACGGCGAGTGGCGAGTTTTAGAGCTAGAAATAGCAAGTTAA |
| PGRB-*yghX*-A | TCGCCACTCGCCGTGCCACAACTAGTATTATACCTAGGACTGAGC |
| Bsu-pyr-1-trc-S | TCCGGCTCGTATAATGTGTGGAATTGTGAGCGGATAACAATTTCACACAGGAAACAGACCATGAAGCATTTAACGACGATGAGT |
| Bsu-pyr-1-4#-A | AGTTGCTGGATTACTATGACCCTAGAAGAAATCAACCAGCGCATCAGAAAGTCTCCTGTGCATGAGCGGAGGATTCATTTTATAGTTT |
| Bsu-pyr-2-S | AAAGCGGGAATCAGAGTGACA |
| Bsu-pyr-2-1#-A | GCCCCAAGGGGTTATGCTAGCCTACAAATTGAGTTATGTTCATTTAAATATGATGTTGTTCAGCCTCCGAGTGTAGGAAGAATGG |
| Bsu-pyr-3-S | TCATCCTTGTCAACTCAAACCCT |
| Bsu-pyr-3-4#-A | AGTTGCTGGATTACTATGACCCTAGAAGAAATCAACCAGCGCATCAGAAAGTCTCCTGTGCATAGGGTGCTGTGGATTGATTTT |
| Bsu-pyr-4-S | GGGCGATGGAGATTGTTTACC |
| Bsu-pyr-4-1#-A | GCCCCAAGGGGTTATGCTAGCCTACAAATTGAGTTATGTTCATTTAAATATGATGTTGTTCAGCCGGCATTCTTCCTCGTAAA |
| Bsu-pyr-5-S | AGGGGTAAATGTCATCCACGTT |
| Bsu-pyr-5-trc-A | ACAGATAAAACGAAAGGCCCAGTCTTTCGACTGAGCCTTTCGTTTTATTTGTTATTTTTTAAACCAATCTTTTGACTCGGGATTTCG |
| *yghE*-U-S | GTCAGGCACTGGCGAAAGAT |
| *yghE*-U-A | AATTGTTATCCGCTCACAATTCCACACATTATACGAGCCGGATGATTAATTGTCAACGCAAGCCATAAACCCACA |
| *yghE*-D-S | CTGGGCCTTTCGTTTTATCTGTTGTTTGTCGGTGAACGCTCTCCTGAGTAGGACAAATTTCCGACATCGAAATGCGT |
| *yghE*-D-A | AGGCGTTGTTGTGGCAGATT |
| *yghE*-4#-S | ATGCACAGGAGACTTTCTGATGCGCTGGTTGATTTCTTCTAGGGTCATAGTAATCCAGCAACTCGGTGAACGCTCTCCTGAGTAGGACAAATTTCCGACATCGAAATGCGT |
| *yghE*-1#-S | CTGAACAACATCATATTTAAATGAACATAACTCAATTTGTAGGCTAGCATAACCCCTTGGGGCCGGTGAACGCTCTCCTGAGTAGGACAAATTTCCGACATCGAAATGCGT |
| PGRB-*yghE-S* | GCTGAAAAAATATCGCCCACGTTTTAGAGCTAGAAATAGCAAGTTAAGG |
| PGRB-*yghE-A* | GTGGGCGATATTTTTTCAGCACTAGTATTATACCTAGGACTGAGC |
| *purR-*QC-1 | ATGGCAACAATAAAAGATGTAGCGAAACGA |
| *purR-*QC-2 | CACAATCCAGCTTTCCGGCTCGACAGATAAGCCCGCTGT |
| *purR-*QC-3 | ACAGCGGGCTTATCTGTCGAGCCGGAAAGCTGGATTGTG |
| *purR-*QC*-4* | TTAACGACGATAGTCGCGGAAC |
| pGRB*-purR*-S | TCCCAATGGTGGTCATGGACGTTTTAGAGCTAGAAATAGCAAGTTAA |
| pGRB*-purR*-A | GTCCATGACCACCATTGGGAACTAGTATTATACCTAGGACTGAGC |
| *yciQ*-U-S | TTACTTGAAGCATTGGGCGAAC |
| *yciQ*-U-A | AATTGTTATCCGCTCACAATTCCACACATTATACGAGCCGGATGATTAATTGTCAACCAGTCAAGATGCCAGGGTTC |
| *yciQ*-D-S | AAAGACTGGGCCTTTCGTTTTATCTGTTGTTTGTCGGTGAACGCTCTCCTGAGTAGGACAAATGTCTGACAAGAACCAGCAAATCCT |
| *yciQ*-D-A | ATAGCTTCACCGTGGGCATAAC |
| PGRB-*yciQ*-S | TACCCGCAATATTAGAGAACGTTTTAGAGCTAGAAATAGCAAGTTAA |
| PGRB-*yciQ*-A | GTTCTCTAATATTGCGGGTAACTAGTATTATACCTAGGACTGAGC |
| *zwf*_bsu_-trc-S | TGGAATTGTGAGCGGATAACAATTTCACACAGGAAACAGACCGTGAAAACAAACCAACAACCAAAAGCA |
| *zwf*_bsu_-trc-A | AACAACAGATAAAACGAAAGGCCCAGTCTTTCGACTGAGCCTTTCGTTTTATTTGTTATATGTTCCACCAGTGTAAGCCGTC |
| *gapC*-U-S | TGGGAAGAAACCACGAAACTCCAT |
| *gapC*-U-A | TGTTATCCGCTCACAATTCCACACATTATACGAGCCGGATGATTAATTGTCAATGTTTCAGCAGGTAGGCGAGAA |
| *gapC*-D-S | GGCCTTTCGTTTTATCTGTTGTTTGTCGGTGAACGCTCTCCTGAGTAGGACAAATAAAACGGTCGCCTGGTACGATAAC |
| *gapC*-D-A | TTATCCGCCGACATTGCTGC |
| PGRB-*gapC*-S | TACCTCCGCCGAGAAATCGCGTTTTAGAGCTAGAAATAGCAAGTTAA |
| PGRB-*gapC*-A | GCGATTTCTCGGCGGAGGTAACTAGTATTATACCTAGGACTGAGC |
| *prs*-trc-UP-S | GTATAATGTGTGGAATTGTGAGCGGATAACAATTTCACACAGGAAACAGACCATGCCTGATATGAAGCTTTTTGCTGG |
| *prs*-UP-A | CGTGCAGAGCCACTGTCAGCACACG |
| *prs*-DW-S | ACAGTGGCTCTGCACGCTGAACAGATTCAG |
| *prs*-DW-trc-A | AAACAACAGATAAAACGAAAGGCCCAGTCTTTCGACTGAGCCTTTCGTTTTATTTGTTAGTGTTCGAACATGGCAGAGATCG |
| *rph*-U-S | ATAGCGCAGGGTACATTCCACTTTAC |
| *rph*-U-A | TTATCCGCTCACAATTCCACACATTATACGAGCCGGATGATTAATTGTCAACCTTCTTCAATAGAGGCGGTACACAA |
| *rph*-D-S | CCTTTCGTTTTATCTGTTGTTTGTCGGTGAACGCTCTCCTGAGTAGGACAAATTGCCGCAGAGACCGACATG |
| *rph*-D-A | ACAGCGGTTGTGGTGGC |
| PGRB-*rph-S* | GGCTGGATCACCGCAGAGTAGTTTTAGAGCTAGAAATAGCAAGTTAA |
| PGRB-*rph*-A | TACTCTGCGGTGATCCAGCCACTAGTATTATACCTAGGACTGAGC |
| *glnA*-trc*-*S | CTCGTATAATGTGTGGAATTGTGAGCGGATAACAATTTCACACAGGAAACAGACCATGTCCGCTGAACACGTACTGA |
| *glnA*-trc-A | CAAACAACAGATAAAACGAAAGGCCCAGTCTTTCGACTGAGCCTTTCGTTTTATTTGTTAGACGCTGTAGTACAGCTCAAACTC |
| *yjiT*-U-S | CGGGTTACGCAGGGAGG |
| *yjiT*-U-A | GGGACTTTGCTGCCTGTTTTTTGACAATTAATCATCCGGCTCGTATAATGTGTGGAATTGTGAGCGGATAACAATT |
| *yjiT*-D-S | CTCGTATAATGTGTGGAATTGTGAGCGGATAACAATTTCACACAGGAAACAGACCATGAGTCTTAACCTGGTAAGTGAACAA |
| *yjiT*-D-A | CTGTTGGCGGTACTGCGTAA |
| PGRB-*yjiT*-S | AAGCACTACCTGTGAAGGGAGTTTTAGAGCTAGAAATAGCAAGTTAA |
| PGRB-*yjiT*-A | GGGGTAAGGACTACGGCTACACTAGTATTATACCTAGGACTGAGC |
| *ppc*-trc*-*S | CGGCTCGTATAATGTGTGGAATTGTGAGCGGATAACAATTTCACACAGGAAACAGACCATGAACGAACAATATTCCGCATTGC |
| *ppc*-trc-A | AACAACAGATAAAACGAAAGGCCCAGTCTTTCGACTGAGCCTTTCGTTTTATTTGTTAGCCGGTATTACGCATACCTGC |
| *yjiK*-U-S | GAATCTCACCTGCGAATGCC |
| *yjiK*-U-A | TATCCGCTCACAATTCCACACATTATACGAGCCGGATGATTAATTGTCAACATCCTGCTGGCAATAAATTGACTCA |
| *yjiK*-D-S | CTGGGCCTTTCGTTTTATCTGTTGTTTGTCGGTGAACGCTCTCCTGAGTAGGACAAATTTGCGATGGATGCCTCTGGTAATATT |
| *yjiK*-D-A | CCTAAGGTCAGAACCAGCATC |
| PGRB-*yjiK*-S | TACGGGGTTTCTCCGCGTTAGTTTTAGAGCTAGAAATAGCAAGTTAA |
| PGRB-*yjiK*-A | TAACGCGGAGAAACCCCGTAACTAGTATTATACCTAGGACTGAGC |
| *aspC*-trc-S | TCCGGCTCGTATAATGTGTGGAATTGTGAGCGGATAACAATTTCACACAGGAAACAGACCATGTTTGAGAACATTACCGCCGC |
| *aspC*-trc-A | CACCGACAAACAACAGATAAAACGAAAGGCCCAGTCTTTCGACTGAGCCTTTCGTTTTATTTGTTACAGCACTGCCACAATCGCT |
| *ydaO*-trc-S | TGGAATTGTGAGCGGATAACAATTTCACACAGGAAACAGACCACAAATCGCTTAATCTGAA |
| *ydaO*-*gfp*-A | ACTCCAGTGAAAAGTTCTTCTCCTTTACTCATTGTGTTCGATG |
| *ydaO*-*gfp*-S | CATCGAACACAATGAGTAAAGGAGAAGAACTTTTCACTGGAGT |
| *gfp*-trc-A | ATAAAACGAAAGGCCCAGTCTTTCGACTGAGCCTTTCGTTTTATTTGCTATTTGTATAGTTCATCCATGCCATGTGTAA |
| pTrc99a-XZ-1 | GGTCTGTTTCCTGTGTGAAATTGTT |
| pTrc99a-XZ-2 | CAAATAAAACGAAAGGCTCAGTCGA |
| *insB*-U-S | GGATGGAGCCTCTGCTTCTGG |
| *insB*-U-A | TGTGTGAAATTGTTATCCGCTCACAATTCCACACATTATACGAGCCGGATGATTAATTGTCAACGGTTGTATGCGCGAGGTTAC |
| *insB*-D-S | AAAGACTGGGCCTTTCGTTTTATCTGTTGTTTGTCGGTGAACGCTCTCCTGAGTAGGACAAATGGTGGAGTTGCATGACAAGGTC |
| *insB*-D-A | GGTGGGCGTGCTGAATAAGAAG |
| *insB*-4#-S | ATGCACAGGAGACTTTCTGATGCGCTGGTTGATTTCTTCTAGGGTCATAGTAATCCAGCAACTGGTGGAGTTGCATGACAAGGTC |
| *insB*-1#-S | CTGAACAACATCATATTTAAATGAACATAACTCAATTTGTAGGCTAGCATAACCCGCTTGGGGGTGGAGCATTGTGACAAGGTC |
| PGRB-*insB*-S | TGGTATTGCGGATGGCGTGTTTTAGAGCTAGAAATAGCAAGTTAA\ |
| PGRB-*insB*-A | GCGCTGACGTGATTTAGCACACTAGTATTATACCTAGGACTGAGC |
| *ydaO-nuoA-N*-A | TGACTTCAGTGGATGTTGACATACTCATTGTGTTCGATG |
| *ydaO-nuoA-N*-1-S | CATCGAACACAATGAGTATGTCAACATCCACTGAAGTCA |
| *nuoA-N*-1-4#-A | GAAGAAATCAACCAGCGCATCAGAAAGTCTCCTGTGCATTGCCCCACTCCAGCG |
| *nuoA-N*-2-S | AGAAACTGGCCGGGATCAC |
| *nuoA-N*-2-1#-A | CAAATTGAGTTATGTTCATTTAAATATGATGTTGTTCAGGGCGTACACCAGCCGC |
| *nuoA-N*-3-S | TGGGAACTGCCGTTCG |
| *nuoA-N*-3-4#-A | GAAGAAATCAACCAGCGCATCAGAAAGTCTCCTGTGCATCCGTCGCTTTCAGCAAAGCT |
| *nuoA-N*-4-S | AAGCCCTGAAAGGTCGCG |
| *nuoA-N*-4-1#-A | CAAATTGAGTTATGTTCATTTAAATATGATGTTGTTCAGCCTGGCGCTTGTATTCCCC |
| *nuoA-N*-5-S | TCGTGGTCGTATCGTTCTGAC |
| *nuoA-N*-5-4#-A | GAAGAAATCAACCAGCGCATCAGAAAGTCTCCTGTGCATCCGTCGTTTGCGGAAGC |
| *nuoA-N*-6-S | TCGGCACTACCGCTGGTCA |
| *nuoA-N*-6-1#-A | CAAATTGAGTTATGTTCATTTAAATATGATGTTGTTCAGCATCCTTGTTGTCGTTATAGCCTTCAAG |
| *nuoA-N*-7-S | TGATCGCACTGCTACCGT |
| *nuoA-N*-7-RBS-A | TTTCAATCTCCTATTATTTCCTTAGAAGCTTACATCAGCGGCATTGCCAAAC |
| RBS-*vgb*_vit_-S | GCTTCTAAGGAAATAATAGGAGATTGAAAATGCTGGATCAGCAGACCATTAAT |
| *vgb*_vit_-trc-A | CGAAAGGCCCAGTCTTTCGACTGAGCCTTTCGTTTTATTTGTTATTCCACTGCCTGTGCATACAGATC |
| *molR*-U-S | GTTGTTTTCTTGCGATTTTGTCTCTCTC |
| *molR*-U-A | AATTGTTATCCGCTCACAATTCCACACATTATACGAGCCGGATGATTAATTGTCAAGAAAGCGGCGATGACTTAACG |
| *molR*-D-S | CCTTTCGTTTTATCTGTTGTTTGTCGGTGAACGCTCTCCTGAGTAGGACAAATCCCGAAAAACCAGAAATTGCAC |
| *molR*-D-A | TTTTGCCCAAGAAGTTCCGTCA |
| *molR*-4#-S | ATGCACAGGAGACTTTCTGATGCGCTGGTTGATTTCTTCTAGGGTCATAGTAATCCAGCAACTCCCGAAAAACCAGAAATTGCAC |
| *molR*-1#-S | CTGAACAACATCATATTTAAATGAACATAACTCAATTTGTAGGCTAGCATAACCCCTTGGGGCCCCGAAAAACCAGAAATTGCAC |
| pGRB-*molR*-S | TGGCATTGCACCTAGCTACTGTTTTAGAGCTAGAAATAGCAAGTTAA |
| pGRB-*molR*-A | AGTAGCTAGGTGCAATGCCAACTAGTATTATACCTAGGACTGAGC |
| *ydaO-cyoA-E*-A | CCCAAACTTTTATTGTATTTCCTGAGTCTCATTGTGTTCGATG |
| *ydaO-cyoA-E*-1-S | CATCGAACACAATGAGACTCAGGAAATACAATAAAAGTTTGGG |
| *cyoA-E*-1-4#-A | GAAGAAATCAACCAGCGCATCAGAAAGTCTCCTGTGCATACGATAAAACCGATGGTCCACAG |
| *cyoA-E*-2-S | AGTTTACATCCTGATCCTGCCTG |
| *cyoA-E*-2-trc-A | AGATAAAACGAAAGGCCCAGTCTTTCGACTGAGCCTTTCGTTTTATTTGTTACCACACAGCAGCCAGCA |
| *nuoA-N*-trc*-*S | ATTGTGAGCGGATAACAATTTCACACAGGAAACAGACCATGAGTATGTCAACATCCACTGAAGTCA |
| *cyoA-E*-trc*-*S | GAGCGGATAACAATTTCACACAGGAAACAGACCATGAGACTCAGGAAATACAATAAAAGTTTGGG |

**Supplementary Table 3** Codon optimized gene sequences in this study

| **Synthetic genes** | **Sequence (5’-3’)** |
| --- | --- |
| *PHM8*_sce_*,* originated from *Saccharomyces cerevisiae*, codon optimized toward *E. coli* | ATGACAATTGCAAAAGATTATAGAACCATATACAGAAACCAAATCAAGAAACAAATCAGACTAAATCAAGAACACTTACAATCTCTCACACACTTAGGCTCCCAAATAAACTTCGAAGTAGACCCACCCAAACTGCCAGACCCGGATCCGGCGCGTAAAGTATTTTTCTTTGATATTGACAATACGTTATATCGCAAGTCTACCAAAGTTCAGCTCTTAATGCAACAGTCGCTGTCAAATTTCTTCAAATACGAACTGGGATTTGATGACGATGAAGCCGAACGTCTGATCGAAAGTTACTACCAGGAATACGGTCTAAGCGTGAAAGGTTTGATCAAAAACAAACAGATCGACGACGTATTACAATATAATACCTTCATTGATGATTCTCTGCCGCTTCAGGACTACCTGAAACCGGACTGGAAACTTCGCGAGCTTCTGATTAACCTGAAGAAGAAAAAGCTGGGCAAATTTGACAAACTGTGGCTGTTTACTAACTCTTATAAAAATCATGCAATTCGGTGCGTTAAAATTCTCGGCATCGCGGATCTGTTCGATGGCATTACGTACTGTCACTATGATCGCCCAATCGAAGAAGAGTTTATCTGCAAGCCCGATCCTAAATTCTTCGAAACCGCCAAACTGCAAAGCGGGCTCAGCAGTTTTGCTAACGCGTGGTTCATCGATGATAACGAATCCAACGTCCGTTCCGCATTGTCCATGGGCATGGGTCATGTGATACACTTGATTGAAGATTATCAGTATGAAAGCGAGAATATTGTGACAAAAGATCATAAAAACAAGCAGCAGTTTTCGATTCTGAAAGACATCCTGGAAATACCGCTGATCATGGACGTTGAGGTCTATCGGCCGAGCAGCATAGCAATTAAAGAAATGGAAGAACTGGAAGAAGAGGGGGAAGCAGTCAACTGGAGTAATCAACAAATCAACGTACAATCATCATAA |
| *vgb*_vit_, originated from *Vitreoscilla*, codon optimized toward *E. coli* | ATGCTGGATCAGCAGACCATTAATATTATTAAAGCAACCGTTCCGGTGCTGAAAGAACATGGCGTGACCATTACCACCACCTTTTATAAAAATCTGTTTGCCAAACATCCGGAAGTTCGTCCGCTGTTTGATATGGGTCGCCAGGAATCTCTGGAACAGCCGAAAGCACTGGCCATGACCGTGTTAGCCGCAGCCCAGAATATTGAAAATTTACCGGCAATTCTGCCGGCCGTGAAAAAAATTGCAGTTAAACATTGTCAGGCAGGTGTGGCCGCCGCACATTATCCGATTGTTGGTCAGGAACTGCTGGGCGCAATTAAAGAAGTGTTAGGCGATGCCGCAACCGATGATATTTTAGATGCATGGGGTAAAGCCTATGGTGTGATTGCAGATGTGTTTATTCAGGTTGAAGCTGATCTGTATGCACAGGCAGTGGAATAA |
| *cipA,* originated from *Photorhabdus luminescens*, codon optimized toward *E. coli* | ATGATTAACGATATGCATCCGAGCCTGATTAAAGATAAAGATATTGTGGATGATGTGATGCTGCGCAGCTGCAAAATTATTGCGATGAAAGTGATGCCGGATAAAGTGATGCAAGTGATGGTGACCGTGCTGATGCATGATGGCGTGTGCGAAGAAATGCTGCTGAAATGGAACCTGCTGGATAACCGCGGCATGGCGATTTATAAAGTGCTGATGGAAGCGCTGTGCGCGAAAAAAGATGTGAAAATTAGCACCGTGGGCAAAGTGGGCCCGCTGGGCTGCGATTATATTAACTGCGTGGAAATTAGCATGTAA |
| *cipB,* originated from *Photorhabdus luminescens*, codon optimized toward *E. coli* | ATGATTATCAAGAAAGATATTCTGCTTAACGAAGAACTTATTGTTGATGACGACTTGAAGGTGGGTAAAGTAGAGAAAGTCAATATTGATATTTTGTCTCCCTCATCGGTTATTGTGTCTTTGAACATCCTTGGTGTGGTGGATGACTTCCATCTGCTTTTGGTAGATGATAAGGATAAAGACAAAATCGTATTGTTGTATCTGTCTCTGCTTCGCGTCCTTCACGAGAAACTGGACGTCAAGGTTAAGGTCGCGAAATCCAAACTTACAAAGATTAAATACATTGTGGGTGTGGAAATCTAA |
